# Recognition of Metal Ion Ligand-Binding Residues by Adding Correlation Features and Propensity Factors

**DOI:** 10.3389/fgene.2021.793800

**Published:** 2022-01-04

**Authors:** Shuang Xu, Xiuzhen Hu, Zhenxing Feng, Jing Pang, Kai Sun, Xiaoxiao You, Ziyang Wang

**Affiliations:** ^1^ College of Sciences, Inner Mongolia University of Technology, Hohhot, China; ^2^ Inner Mongolia Key Laboratory of Statistical Analysis Theory for Life Data and Neural Network Modeling, Hohhot, China

**Keywords:** metal ion ligand, binding residues, correlation features, propensity factors, GBM algorithm

## Abstract

The realization of many protein functions is inseparable from the interaction with ligands; in particular, the combination of protein and metal ion ligands performs an important biological function. Currently, it is a challenging work to identify the metal ion ligand-binding residues accurately by computational approaches. In this study, we proposed an improved method to predict the binding residues of 10 metal ion ligands (Zn^2+^, Cu^2+^, Fe^2+^, Fe^3+^, Co^2+^, Mn^2+^, Ca^2+^, Mg^2+^, Na^+^, and K^+^). Based on the basic feature parameters of amino acids, and physicochemical and predicted structural information, we added another two features of amino acid correlation information and binding residue propensity factors. With the optimized parameters, we used the GBM algorithm to predict metal ion ligand-binding residues. In the obtained results, the Sn and MCC values were over 10.17% and 0.297, respectively. Besides, the S_n_ and MCC values of transition metals were higher than 34.46% and 0.564, respectively. In order to test the validity of our model, another method (Random Forest) was also used in comparison. The better results of this work indicated that the proposed method would be a valuable tool to predict metal ion ligand-binding residues.

## 1 Introduction

The realization of protein functions requires interaction with ligands; in particular, metalloproteins formed by the combination of proteins and metal ion ligands play a vital role in biological functions (Barondeau and Getzoff, 2004). For example, the binding of Cu^2+^ ligand can promote *in situ* oxidation modification reaction (Cecconi et al., 2002), and the oxygen-promoting compound formed by the combination of Mn^2+^ ligands and proteins can be used as a catalyst in the process of photosynthesis (Reed and Poyner, 2000). In fact, the mechanism of protein–metal ion ligand binding is that some special protein functions need the precise binding of proteins and ligand-binding residues, while the abnormal binding would lead to many related diseases. For example, abnormal binding residues of Cu^2+^ ligand can lead to the diseases of Wilson and Menkes (Yuan et al., 1995; Petris et al., 1996). In addition, metal ions have a direct influence on the formation of Alzheimer’s and Parkinson’s diseases (Barnham and Bush, 2008). Therefore, the study of protein–metal ion ligand-binding residues is helpful to understand the mechanism of protein functions, the treatment of diseases, and the design of molecular drugs.

Many reported literatures showed that the appropriate feature parameters were the basis of recognizing metal ion ligand-binding residues (Horst and Samudrala, 2010; Lu et al., 2012; Yang et al., 2013a; Jiang et al., 2016; Cao et al., 2017; Wang et al., 2020). For example, in 2010, Horst and Samudrala (2010) extracted amino acids, local conservatism, and other features of Ca^2+^ ligand in prediction, and Matthew’s correlation coefficient (MCC) was up to 0.6. In 2012, Lu et al. (2012) adopted a method of fragment conversion, and the prediction accuracy (ACC) of 6 ligands reached 94.6%. In 2016, Jiang et al. (2016) used the information of increment of diversity, matrix score, and autocross covariance as prediction parameters, the ACC values of the Ca^2+^ ligand exceeded 75.0%, and the MCC value exceeded 0.50. In 2017, Cao et al. (2017) extracted the component and site-conserved information of amino acids, physicochemical features, and structural information, the ACC values were higher than 74.8%, and the MCC values were higher than 0.5.

In terms of algorithms, many machine learning algorithms were used in the recognition of metal ion ligand-binding residues (Hu et al., 2016a; Hu et al., 2016b; Liu et al., 2019; Wang et al., 2019; Liu et al., 2020). For example, in 2016, Hu et al. (2016a) used SVM algorithm and the 9 metal ion ligands; Ionseq obtained good prediction results. In 2019, Wang et al. (2019) applied the SMO algorithm to predict 10 metal ion ligand-binding residues and obtained better prediction results. In 2019, Liu et al. (2019) applied the K-nearest neighbor classifier, and the ACC values of 6 metal ion ligands were higher than 80.0%. In 2020, Liu et al. (2020) used Random Forest (RF) algorithm in predicting the 10 kinds of ion binding residues, and the MCC values were higher than 0.55.

In the prediction works of metal ion ligands, many researchers found several important feature parameters such as amino acid, secondary structure, relative solvent accessibility, hydrophilic–hydrophobic, and polarization charge at the fragment level. In this study, through the statistical analysis for the correlation of amino acids, we found that there exists a high probability of the occurrence of the adjacent, secondary neighbor, and thirdly neighbor of the binding residues. Therefore, we took the amino acid correlation information of amino acids into consideration when extracting feature parameters. In addition, because the binding of metal ion ligands to specific amino acids residues has a certain tendency, we counted the difference between non-binding residues and binding residues bound by different metal ions. Thus, we further took the binding residue propensity factors as feature parameters. In the datasets of this work, the serious imbalance of the positive and negative sets would result in a high false positive in the prediction results. In this study, we chose the GBM (Gradient Boostling) algorithm, which has a comparative advantage in the above problem. The algorithm can optimize the model by continuously reducing the sample errors and improve the prediction overall accuracy by optimizing the algorithm parameters in the prediction.

## 2 Materials and Methods

### 2.1 Dataset

In this paper, 10 kinds of metal ion ligand-binding residues were studied. In order to ensure the authenticity and reliability of the experimental data source, the datasets constructed by our group (Cao et al., 2017) were from the semi-manual Biolip database (Yang et al., 2013b), which was measured by experiments with high accuracy. The 10 metal ions in the datasets contain Zn^2+^, Cu^2+^, Fe^2+^, Fe^3+^, Co^2+^, Mn^2+^, Ca^2+^, Mg^2+^, Na^+^, and K^+^. In the datasets, the arbitrary protein sequence was longer than 50 amino acids. In addition, the resolution and sequence identity thresholds were lower than 3 Å and 30%, respectively.

Since the surrounding residues also have an influence on the binding of metal ion ligands, we considered the binding residues and surrounding residues in the datasets. In the work, we used the sliding window method to intercept fragments from the beginning of the protein chains. To ensure that each amino acid can appear in the center of a fragment, we added (L−1)/2 pseudo-amino acids to both ends of a protein chain, in which the pseudo-amino acid was represented by X. If the central position of one fragment was a binding residue, then we defined the fragment as a positive sample; otherwise, it was a negative one. The datasets are shown in Table 1. According to the physicochemical properties of ions, we also divided the 10 metal ion ligands into 3 categories: transition-metal ions (Zn^2+^, Cu^2+^, Fe^2+^, Fe^3+^, Co^2+^, and Mn^2+^), alkaline-earth metal ions (Ca^2+^ and Mg^2+^), and alkali-metal ions (Na^+^ and K^+^).

**TABLE 1:** The benchmark datasets of ten metal ion ligands.

Metal ion ligand	Chains	P	N	Metal ion ligand	Chains	P	N
Zn^2+^	1,428	6,408	405,113	Mn^2+^	459	2,124	156,625
Cu^2+^	117	485	33,947	Ca^2+^	1,237	6,789	396,957
Fe^2+^	92	382	29,345	Mg^2+^	1,461	5,212	480,307
Fe^3+^	217	1057	68,829	Na^+^	78	489	27,408
Co^2+^	194	875	55,050	K^+^	53	535	18,777

The second column is the number of protein chains; P is the number of binding residues; N is the number of non-binding residues.

### 2.2 Selection and Extraction of Feature Parameters

#### 2.2.1 Basic Features Parameters

**FIGURE 1:**
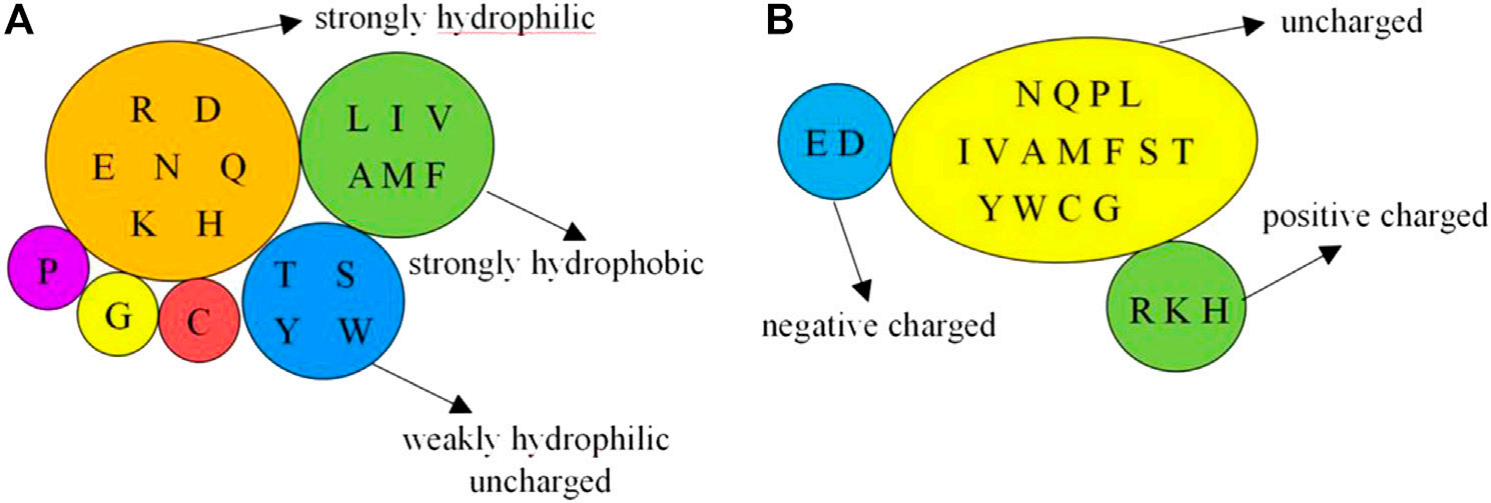
Classification of physicochemical features of amino acids. Note: **(A)** is 6 categories of the hydrophilic–hydrophobic; **(B)** is 3 categories of the polarization charge.

On the basis of the primary sequence of the protein, we selected the amino acids, and physicochemical and predicted structural information as basic feature parameters. These parameters have been widely used in previous works (Hu et al., 2016a; Cao et al., 2017; Liu et al., 2019; Wang et al., 2019; Liu et al., 2020; Wang et al., 2020). The physicochemical features contain hydrophilic–hydrophobic and polarization charge information. According to the hydrophilic–hydrophobic of amino acids (Pánek et al., 2005), we divided the 20 amino acids into 6 categories. Depending on the charged condition of amino acids after the hydrolysis, we divided the 20 amino acids into 3 categories (Taylor, 1986). The detailed classification is presented in Figure 1.

By using the ANGLOR software (Wu and Zhang, 2008), we obtained the predicted structural features including secondary structure and relative solvent accessibility from the primary sequence of protein. Here, we divided the secondary structure into three categories: α-helix, β-sheet, and coil. In addition, we divided the relative solvent accessibility into two categories: exposed and buried. If the Boolean values of amino acid were larger than 0.25, then the amino acids were defined as “exposed" ones; otherwise, they were defined as “buried” ones.

#### 2.2.2 Amino Acid Correlation Features

We took a detailed statistical analysis for the correlation features of amino acids. According to the analysis results, we calculated the correlation information of amino acids; the detailed steps were as follows:

##### 2.2.2.1 Sequence-Based Correlation Statistical Analysis

**FIGURE 2:**
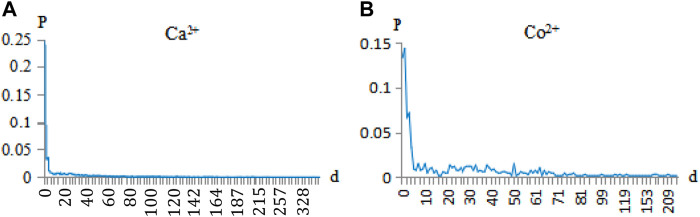
Correlation probability of Ca^2+^and Co^2+^ ligand-binding residues. Note: the abscissa d is the correlation of the binding residues (e.g., d = 0 is the adjacent, d = 1 is the secondary neighbor, d = 2 is the thirdly neighbor). The ordinate p is the probability of correlation between the binding residues.

Due to protein folding in the 3D structure, one spatial binding site of a metal ion ligand usually refers to several surrounding binding residues. In this way, although the spatial distance of these surrounding residues is very close, the sequence distance may be very long. For example, on the BS01 binding site of the protein (3I11A), the binding residues bound with Co^2+^ ligands were located at 86, 88, 90, and 149 positions in the same sequence, respectively. These binding residues may have long-range correlation (Chen et al., 2018; Zhang et al., 2020). Then, for every protein chain, we scanned from the first binding residue and counted the distance between the two binding residues sequentially. Taking Ca^2+^ and Co^2+^ ligands as examples, the binding residues are shown in Figure 2.

In Figure 2, the correlations of the adjacent, secondary neighbor and thirdly neighbor between binding residues accounted for a large proportion. Since the occurrence probability of *d* > 6 is not high, we showed the probability of *d* < 6 for the 10 metal ions in Table 2.

**TABLE 2:** The correlation probability of 10 metal ion ligand-binding residues.

Ligands	d = 0	d = 1	d = 2	d = 3	d = 4	d = 5	d = 6
Zn^2+^	0.040	0.120	0.184	0.082	0.046	0.022	0.016
Cu^2+^	0.087	0.180	0.071	0.087	0.082	0.016	0.011
Fe^2+^	0.028	0.190	0.087	0.066	0.024	0.017	0.014
Fe^3+^	0.082	0.126	0.105	0.072	0.017	0.018	0.006
Co^2+^	0.134	0.144	0.066	0.072	0.032	0.009	0.007
Mn^2+^	0.150	0.161	0.058	0.041	0.016	0.005	0.011
Ca^2+^	0.247	0.240	0.097	0.032	0.035	0.012	0.008
Mg^2+^	0.216	0.165	0.090	0.048	0.016	0.007	0.006
Na^+^	0.434	0.139	0.080	0.017	0.005	0.010	0.007
K^+^	0.547	0.108	0.035	0.025	0.008	0.010	0.010

From Table 2, we found that the probabilities of the adjacent, secondary neighbor, and thirdly neighbor correlations for the ten ions were different. For a metal ion ligand, we selected the correlation information with probability >10% to extract parameters. In this way, for Co^2+^, Mn^2+^, Ca^2+^, Mg^2+^, Na^+^, and K^+^, we extracted the adjacent and secondary neighbor correlation information. For Zn^2+^ and Fe^3+^, we extracted the secondary neighbor and thirdly neighbor correlation information. For Fe^2+^ and Cu^2+^, we extracted the second-neighbor correlation information.

##### 2.2.2.2 Further Screening of Related Features

The probability of the occurrence of 400 pairs of amino acids in the positive and negative sets of each ion ligand was counted separately. We used vector *B* to represent 20 kinds of amino acids and then made a 20*20 matrix *J* for the 400 pairs of amino acids. The matrix *J* of the pairs of amino acid was defined as follows:

$$J=B^{T}B={\left(\begin{array}{c} A\\ C\\ D\\ \vdots \\ W\\ Y \end{array}\right)}_{20\times 1}{\left(\begin{array}{cccccc} A & C & D & \cdots & W & Y \end{array}\right)}_{1\times 20}={\left(\begin{array}{cccccc} AA & AC & AD & \cdots & AW & AY\\ CA & CC & CD & \cdots & CW & CY\\ DA & DC & DD & \cdots & DW & DY\\ \vdots & \vdots & \vdots & \ddots & \vdots & \vdots \\ WA & WC & WD & \cdots & WW & WY\\ YA & YC & YD & \cdots & YW & YY \end{array}\right)}_{20\times 20}$$

**FIGURE 3:**
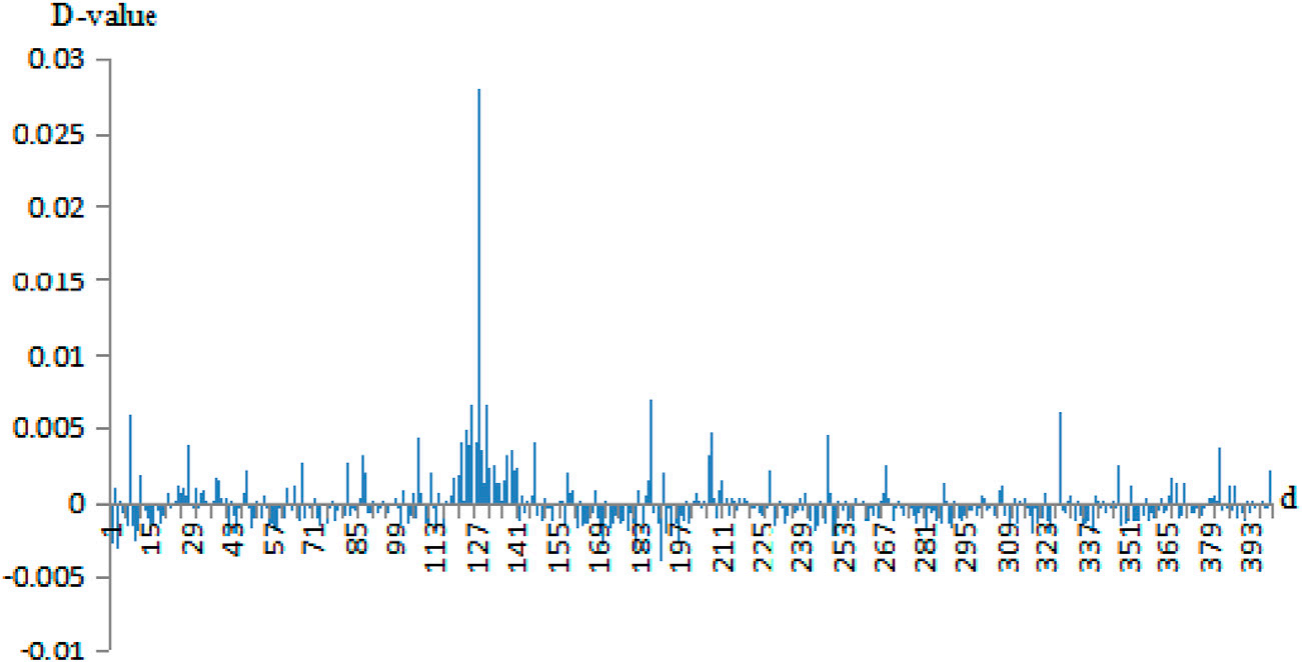
The probability difference of the secondary neighbor correlation of Cu^2+^ positive and negative fragments. Note: the abscissa 1–400 is AA, AC, AD, …, AY, CA, CC, CD, …, CY, …, YA, YC, YD, …, YY. The ordinate is the D-values of the positive sets minus the negative sets.

**FIGURE 4:**
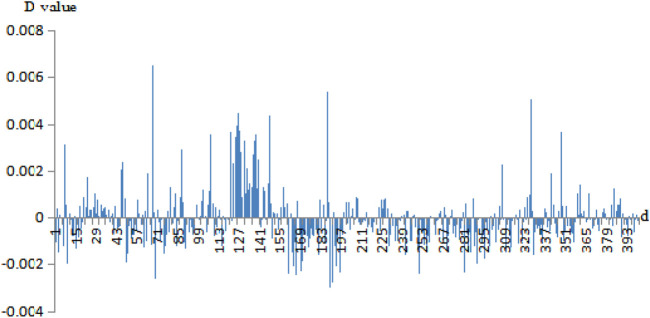
The probability difference of the thirdly neighbor correlation of Fe^3+^ positive and negative fragments. Note: the abscissa 1–400 is AA, AC, AD, …, AY, CA, CC, CD, …, CY, …, YA, YC, YD, …, YY. The ordinate is the D-values of the positive sets minus the negative sets.

Then, we calculated the D-values of the probability of 400 pairs of amino acids between the negative sets and the positive sets. For example, the D-value differences of correlation information of Cu^2+^ secondary neighbor and Fe^3+^ thirdly neighbor are given in Figures 3, 4, respectively.

In Figures 3, 4, the abscissa was the 400 amino acid pairs from matrix *J*, the corresponding vector (AA, AC, AD, …, AY, CA, CC, CD, …, CY, …, YA, YC, YD, …, YY). The ordinate was the D-values between the positive sets and the negative sets. In Figure 4, If the bars were above the *x*-axis, it represents that the occurrence probability of amino acids pairs of the positive sets was greater. Otherwise, the probability of the negative sets was greater. In Figure 3, the abscissa values of Cu^2+^ secondary neighbor correlation were 7, 127, 187, and 327; the corresponding AH, HH, LH, and TH pairs of amino acids had a great difference in probability between positive and negative sets. They tended to appear in positive sets; in particular, the HH had a larger difference in probability. In Figure 4, the abscissa values of the Fe^3+^ thirdly neighbor correlation were 67, 126, 147, 187, 327, and 347 corresponding to EH, HG, IH, LH, TH, and VH. They had great probability differences between the positive and negative sets, and preferred to appear in positive sets. Among them, EH, LH, and TH were more obvious. The probability difference of EK, LK, LL, and RA between the positive and negative sets was greater, and these pairs preferred to appear in negative sets.

##### 2.2.2.3 Feature Parameters of Amino Acid Correlation

Due to the fact that the 400 pairs of amino acids appear differently between positive and negative sets, the ones with little difference would cause information redundancy of prediction parameters. Therefore, we sorted the absolute values of the probability difference in descending order obtained from the top 100 features. Then, we divided them into 10 groups in order. Within each group, there were 10 features. Finally, we took the amino acid correlation features as feature parameters.

#### 2.2.3 Binding Residues Propensity Factors

**FIGURE 5:**
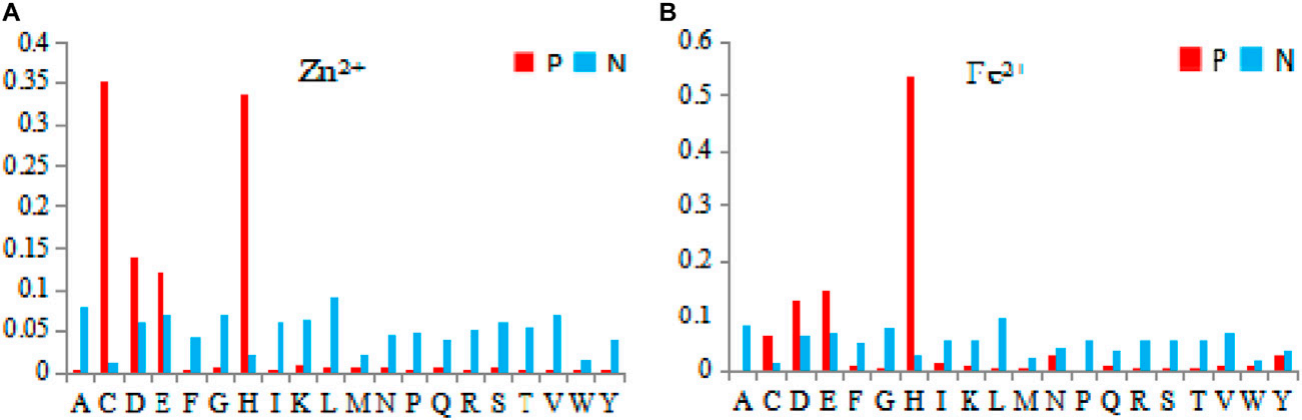
The probability of 20 amino acids in Zn^2+^ and Fe^2+^ bounding residues. Note: The abscissa values represents 20 amino acids, the letters of an alphabet in ordinate represents the probability. P represents the binding residue, and N represents the non-binding residue.

Previous studies on predicting the ligand-binding residues were usually based on the binding residues and their surrounding residues. However, the features of the binding residues alone were not taken into consideration. In fact, the ligand-specific binding also has a selective preference for different amino acid residues. Therefore, we counted the amino acid residues that the 10 metal ion ligands preferred to bind. For example, Zn^2+^ and Fe^2+^ are shown in Figure 5.

In Figure 5, among the 20 amino acids, the four amino acids of C, D, E, and H were more likely to be the binding residues. However, for Zn^2+^ and Fe^2+^ ligands, the four amino acids were used differently. In comparison, C and H were more easily bound by Zn^2+^ ligands, while H was more easily bound by Fe^2+^ ligands. Therefore, we extracted propensity factor of binding residues as feature parameters. The formula of the propensity factor (Chou and Fasman, 1974) was as follows:

$$F_{ij}=\frac{p_{ij}}{p_{j}}$$ (1)

The statistical samples were binding residues and non-binding residues, $p_{ij}=\frac{n_{ij}}{N_{i}}$ , $p_{j}=\frac{N_{j}}{N_{t}}$ ; i is 20 amino acids (*i* = 1,2, … 20); j is binding residues or non-binding residues (*j* = 1,2); $n_{ij}$ represents the number of amino acid i in binding residues or non-binding residues; $N_{i}$ represents the number of amino acid i in the statistical samples; $N_{j}$ represents the number of binding or non-binding residues; $N_{t}$ represents the number of residues in the statistical samples. If $F_{ij}$ is larger than 1, it means that type amino acid i is more inclined to be amino acid j . Taking Mn^2+^ as an example, the values of propensity factor of amino acids D, E, H, and N were larger than 1, indicating that the 4 amino acids were more likely to become binding residues (Table 3).

**TABLE 3:** The binding and non-binding residue amino acid propensity factors of Mn^2+^.

	*F*_*p*_	*F*_*n*_	—	*F*_*p*_	*F*_*n*_
A	0.1691	1.0113	M	0.1871	1.011
C	0.8155	1.0025	N	1.0771	0.999
D	5.0717	0.9448	P	0.0799	1.0125
E	2.5358	0.9792	Q	0.397	1.0082
F	0.3309	1.0091	R	0.4359	1.0076
G	0.2841	1.0097	S	0.4785	1.0071
H	9.1739	0.8892	Y	0.4109	1.008
I	0.1966	1.0109	V	0.1349	1.0117
K	0.5819	1.0057	W	0.2386	1.0103
L	0.0599	1.0127	Y	0.4079	1.008

*F*_*p*_ is the propensity of binding residues; *F*_*n*_ is the propensity of non-binding residues.

#### 2.2.4 Extraction of Feature Parameters

Besides the propensity factors for feature parameters, we also used components, matrix scoring, and information entropy to extract parameters. First, the component information of amino acids, correlation features, secondary structure, and relative solvent accessibility were extracted. Then, the position weight matrix was used to extract the conservative information of the site as a predictive parameter (Hu et al., 2016a; Liu et al., 2019; Wang et al., 2019; Liu et al., 2020; Wang et al., 2020). In this paper, based on the above matrix, the 2L-dimensional site conservative information of amino acids, secondary structure, and relative solvent accessibility were obtained. The position weight matrix formula was as follows:

$$m_{i,j}=\ln\left(\frac{p_{i,j}}{p_{0,j}}\right)$$ (2)

$$p_{i,j}=\frac{n_{i,j}+\frac{\sqrt{N_{i}}}{q}}{N_{i}+\sqrt{N_{i}}}$$ (3)

Where i denotes the site, j represents 20 amino acids and pseudo-amino acid X, $P_{i,j}$ represents the probability of occurrence of amino acid sites at the *i*th position, and $P_{0,j}$ represents the background probability. $n_{i,j}$ represents the number of amino acids *j* at the *i*th position, $N_{i}$ represents the number of all amino acids at the *i*th position, and *q* represents the number of categories *q* = 21. Two scoring matrices can be obtained by using positive and negative training sets, and a 2L (L is the window length)-dimensional feature vector can be obtained for arbitrary fragment. Similarly, for the secondary structure (*q* = 4) and relative solvent accessibility (*q* = 3), 2L-dimensional site conservation features can also be obtained.

As the number of amino acids included in the classification of the hydrophilic–hydrophobic and polarized charges of amino acids was not uniform, information entropy (Liu et al., 2020; Wang et al., 2020) was used to extract the hydrophilic–hydrophobic and polarized charges. The formulas for information entropy were as follows:

$$H\left(x\right)=-\overset{q}{\Sigma }p_{j}\log_{2}p_{j}$$ (4)

$$p_{j}=\frac{\left(n_{j}+\frac{\sqrt{N}}{q}\right)}{\left(N+\sqrt{N}\right)}$$ (5)

Where *j* = 1, 2, … *q*, *q* represents the number of categories, $N=\sum_{j=1}^{q}n_{j}$ , *n*_*j*_ represents the frequency of occurrence of hydrophilic–hydrophobic or polarized charges in the classification, and *p*_*j*_ represents the probability of occurrence of a certain category, hydrophilic–hydrophobic (*q* = 7) and polarized charge (*q* = 4). For arbitrary fragment, one-dimensional hydrophilic–hydrophobic information entropy and one-dimensional polarization charge information entropy can be obtained.

### 2.3 Gradient Boosting Machine Algorithm

As an improved Boosting algorithm, GBM algorithm was proposed by Friedman (2001). It achieved excellent results in many data mining competitions and was widely used in many fields (Feng and Li, 2017; Rawi et al., 2017; Hu et al., 2020). The advantage of the GBM is that it inherits the advantages of a single decision tree and discards its shortcomings. It can fit complex nonlinear relationships with fast calculation speed, strong robustness, and high accuracy. The deviation of the model will not have a serious impact on the algorithm. The GBM improves the model by adding a new classifier to continuously decrease the overall residual; after the iteration, the classifier is as follows:

$$F_{m}\left(x\right)=F_{m-1}\left(x\right)+\rho_{m}h_{m}\left(x\right)$$ (6)

Where *m* is the number of iterations, $\rho_{m}$ is the weight value (the distance the loss function drops in its gradient direction), and $h_{m}\left(x\right)$ is the fitting function of the sample residuals $y-F_{m-1}\left(x\right)$ in the iteration process.

This article used the “gbm” package in R software version 3.6.3. Here, in the algorithm, we mainly optimized the four adjustable parameters (i.e., n.trees, interaction.depth, shrinkage, and n.minobsinnode) (Rawi et al., 2017; Hu et al., 2020).

### 2.4 The Validation Methods and Evaluation Metrics

The 5-fold cross-validation was generally used to identify binding residues (Hu et al., 2016a; Hu et al., 2016b; Liu et al., 2019; Wang et al., 2019; Liu et al., 2020; Wang et al., 2020). The following 4 evaluation indicators were used to evaluate the recognition ability of the prediction model (Jiao and Du, 2016; Chen et al., 2019): sensitivity (S_n_), specificity (S_p_), accuracy (Acc), and Matthew’s correlation coefficient (MCC). The formulas were defined as follows:

$$S_{n}=\frac{TP}{TP+FN}\times 100\%$$ (7)

$$S_{p}=\frac{TN}{TN+FP}\times 100\%$$ (8)

$$Acc=\frac{TP+TN}{TP+TN+FP+FN}\times 100\%$$ (9)

$$MCC=\frac{\left(TP\times TN\right)-\left(FP\times FN\right)}{\sqrt{\left(TP+FP\right)\left(TP+FN\right)\left(TN+FP\right)\left(TN+FN\right)}}$$ (10)

In the above formulas, TP is the number of correctly predicted binding residues, FN is the number of incorrectly predicted binding residues, TN is the number of correctly predicted non-binding residues, and FP is the number of incorrectly predicted non-binding residues.

## 3 Calculation Results and Discussion

### 3.1 The Prediction Framework

**FIGURE 6:**
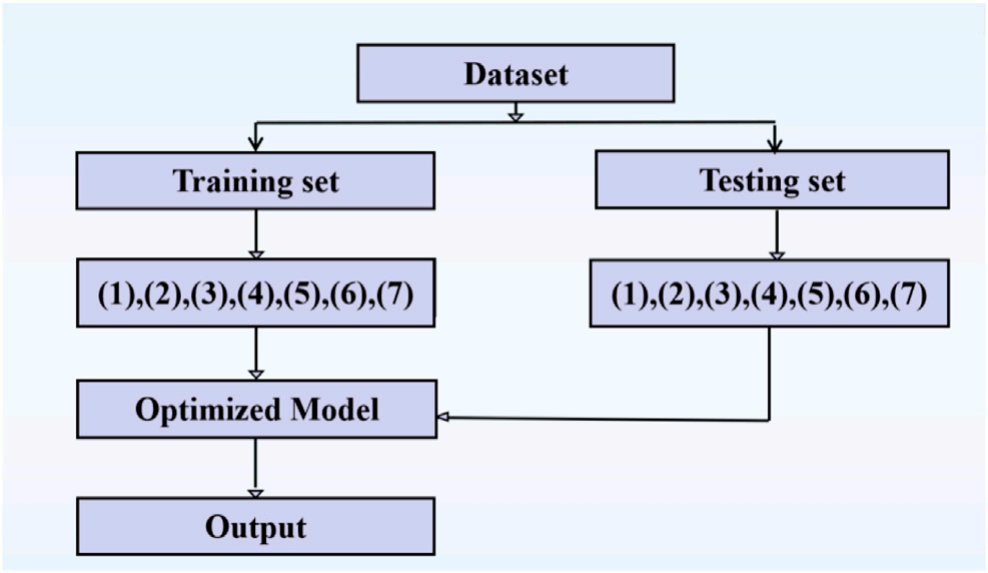
The work flow of identifying the ion ligand binding sites. Note: (1), (2), (3), …, (7) represent the different types of features.

The prediction parameters from Sections 2.2.3, 2.2.4 are summarized and shown in Table 4. The work flow of identifying the ion ligand binding sites is shown in Figure 6.

**TABLE 4:** A summary of prediction parameters.

Features	Extraction of feature parameters and dimensions
Amino acid	(1) amino acid: 21-dimensional component information + 2L-dimensional position conservation information
Structure	(2) secondary structure: 4-dimensional component information + 2L-dimensional position conservation information
—	(3) relative solvent accessibility: 3-dimensional component information + 2L-dimensional position conservation information
Physicochemical	(4) hydrophilic–hydrophobic: 1-dimensional entropy value
—	(5) charge: 1-dimensional entropy value
Two feature parameters	(6) correlation features: 20-dimensional component information (Fe^2+^ and Cu^2+^ correlation features are 10-dimensional)
—	(7) 2-dimensional binding residue propensity factors

### 3.2 Results and Discussion

In prediction, we used the full parameters of Table 5 and input the combined features into the GBM algorithm. Then, we calculated the results of 7 window lengths (i.e., 5, 7, 9, 11, 13, 15, and 17) on a 5-fold cross-validation test. In the process, we defined the corresponding window lengths as the optimal ones (L) with higher S_n_ and MCC values. The predicted results of GBM^(1)^ with the optimal window are shown in Table 5.

**TABLE 5:** Comparison of 5-fold cross-validation results.

Ligands	Method	L	S_n_ (%)	S_p_ (%)	Acc (%)	MCC
Zn^2+^	GBM^(1)^	11	29.82	99.85	98.76	0.473
	GBM^(2)^	11	38.17	99.90	98.94	0.570
	RF	11	39.18	99.77	98.83	0.531
	Ionseq	13	43.56	99.57	99.21	0.504
Cu^2+^	GBM^(1)^	15	40.82	99.86	99.03	0.570
	GBM^(2)^	15	59.38	99.95	99.38	0.747
	RF	15	33.20	99.83	98.89	0.488
	Ionseq	15	50.65	99.69	99 0.01	0.587
Fe^2+^	GBM^(1)^	13	37.17	99.85	99.04	0.527
	GBM^(2)^	13	55.50	99.92	99.35	0.705
	RF	13	21.20	99.88	98.87	0.383
	Ionseq	9	54.08	99.51	98.84	0.577
Fe^3+^	GBM^(1)^	15	18.45	99.86	98.63	0.349
	GBM^(2)^	15	44.75	99.93	99.10	0.634
	RF	15	27.25	99.78	98.69	0.420
	Ionseq	11	55.27	99.81	99.21	0.637
Co^2+^	GBM^(1)^	11	12.69	99.94	98.57	0.308
	GBM^(2)^	11	43.54	99.95	99.06	0.632
	RF	11	12.77	99.81	98.45	0.252
	Ionseq	—	—	—	—	—
Mn^2+^	GBM^(1)^	13	9.60	99.93	98.73	0.249
	GBM^(2)^	13	34.46	99.97	99.09	0.564
	RF	13	16.62	99.82	98.71	0.299
	Ionseq	11	31.07	99.82	99.01	0.455
Ca^2+^	GBM^(1)^	13	3.79	99.97	98.36	0.161
	GBM^(2)^	13	10.75	99.97	98.47	0.302
	RF	13	6.94	99.75	86.21	0.214
	Ionseq	9	22.72	99.04	98.18	0.211
Mg^2+^	GBM^(1)^	15	1.80	99.99	98.92	0.108
	GBM^(2)^	15	10.17	99.98	99.02	0.297
	RF	15	7.12	99.96	98.96	0.214
	Ionseq	15	5.57	99.98	99.49	0.183
Na^+^	GBM^(1)^	13	8.38	99.96	98.35	0.254
	GBM^(2)^	13	16.97	99.97	98.52	0.392
	RF	13	0.2	100	98.25	0.045
	Ionseq	13	77.14	74.04	74.09	0.152
K^+^	GBM^(1)^	13	7.28	99.98	97.41	0.253
	GBM^(2)^	13	25.61	99.96	97.90	0.488
	RF	13	0.93	100	97.26	0.095
	Ionseq	11	8.52	99.88	97.32	0.228

L is the optimal window; GBM^(1)^ is the result of the default setting of the GBM, algorithm parameters; GBM^(2)^ is the result of optimizing the GBM, algorithm parameters.

In the results of GBM^(1)^ (Table 5), the predicted results of transition-metal ion ligands were better. The S_n_ and MCC values of Zn^2+^, Cu^2+^, and Fe^2+^ ligands were higher than 29.82% and 0.473, respectively. The S_n_ and MCC values of Fe^3+^, Co^2+^, and Mn^2+^ ligands were higher than 9.6% and 0.249, respectively. The S_n_ and MCC values of alkali–metal ion ligands were higher than 7.28% and 0.253, respectively.

In order to test the validity of the amino acid correlation information and binding residue propensity factor, we removed correlation features or propensity factors from the full feature sets. Taking Cu^2+^ and Na^+^ ligands as examples, the results are shown in Table 6.

**TABLE 6:** The results of 5-fold cross-validation.

Ligand	Method	S_n_ (%)	S_p_ (%)	Acc (%)	MCC
Cu^2+^	(a)	29.28	99.85	98.86	0.461
	(b)	31.13	99.85	98.88	0.479
	(c)	39.38	99.85	99.00	0.533
	(d)	40.82	99.86	99.03	0.570
Na^+^	(a)	1.84	99.99	98.27	0.116
	(b)	7.16	99.96	98.33	0.228
	(c)	5.32	99.97	98.32	0.202
	(d)	8.38	99.96	98.35	0.254

The prediction parameter of (a) is (1)+(2)+(3)+(4)+(5); the prediction parameter of (b) is (1)+(2)+(3)+(4)+(5)+(6); the prediction parameter of (c) is (1)+(2)+(3)+(4)+(5)+(7); the prediction parameter of (d) is (1)+(2)+(3)+(4)+(5)+(6)+(7).

In comparison with (a), for Cu^2+^ ligand: the S_n_ and MCC values of (b) were higher, and S_n_ and MCC values of (c) increased by 10.1% and 0.072, respectively. When parameters of correlation feature and propensity factor were added, the S_n_ and MCC value were significantly increased by 11.54% and 0.109, respectively. For Na^+^ ligand: the Sn and MCC values of (b) were significantly improved by 5.32% and 0.112, respectively. The Sn and MCC values of (c) were increased. When correlation feature and propensity factor were added, the S_n_ and MCC values increased by 6.54% and 0.138, respectively.

On the addition of feature parameters, different metal ion ligands have different sensitivities. For instance, the Cu^2+^ ligand was more sensitive to the propensity factor, while the Na^+^ ligand was more sensitive to the correlation feature. Above all, the results of adding two parameters were better than those of adding one alone.

**FIGURE 7:**
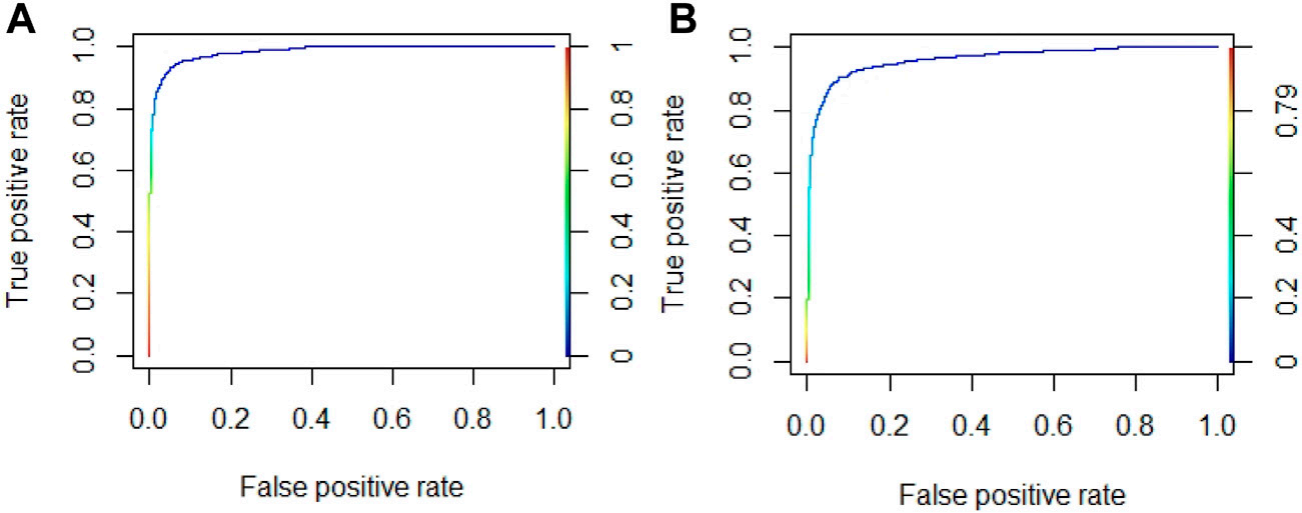
The ROC curve of optimal algorithm parameters for Cu^2+^and K^+^ ligands. Note: **(A)** is Cu^2+^ ligand; **(B)** is K^+^ ligand.

In order to further improve the prediction accuracy, we optimized the four parameters (e.g., n.trees, interaction.depth, shrinkage, and n.minobsinnode) in the GBM algorithm. According to the reported literature (Rawi et al., 2017; Hu et al., 2020), the parameter range was set as follows: n.trees in n{100,150,200,250,300,350,400,450,500}, interaction.depth in d{3,5,7,9}, shrinkage in r{0.01,0.1}, and n.minobsinnode in m{10,20,30,40,50}. The AUROC values were used as the evaluation indicator to obtain the optimal algorithm parameters by the grid search method. Taking Cu^2+^ and K^+^ ligands as examples, the optimal parameters of Cu^2+^ ligand were (5,250,0.1,40), and the AUROC value was 0.985. The optimal parameters of K^+^ ligand were (9,200,0.1,10), and the AUROC value was 0.963. The ROC curves corresponding to the optimal parameters of Cu^2+^ and K^+^ ligands are shown in Figure 7.

As can be seen in Figure 6, the AUROC values of Cu^2+^ and K^+^ ligands both exceed 0.96. For the convenience of comparison, the results after optimizing the algorithm parameters were also added in Table 6.

From the results of GBM^(2)^ in Table 6, it can be seen that the values of S_n_ and MCC of transition metal ion ligands were higher than 34.46% and 0.564, respectively. The values of S_n_ and MCC in the results of alkaline Earth metal ion ligands were higher than 10.17% and 0.297, respectively. The values of S_n_ and MCC in the results of alkali metal ion ligands were higher than 16.97% and 0.392, respectively. In comparison with the results of GBM^(1)^, the results of GBM^(2)^ were significantly improved, in which the S_n_ and MCC values of the nine ligands (i.e., Cu^2+^, Fe^2+^, Fe^3+^, Co^2+^, Mn^2+^, Ca^2+^, Mg^2+^, Na^+^, and K^+^) increased by more than 6.96% and 0.141, respectively.

To verify the stability of those parameters in prediction, the Random Forest (RF) algorithm was also used on the same parameters. The number of decision trees in the RF was set as 500 (Liaw and Wiener, 2002; Liu et al., 2020). The results of the RF were added in Table 6. Except for the alkali metal ion ligands, the S_n_ and MCC values of the other ion ligands were higher than 6.94% and 0.214. The predicted results of transition metal ion ligands were better. The S_n_ and MCC values of Zn^2+^, Cu^2+^, and Fe^3+^ ligands were higher than 27.25% and 0.420, respectively. The S_n_ and MCC values of Fe^2+^, Co^2+^, and Mn^2+^ ligands were higher than 12.27% and 0.252, respectively. Taken together, with the same parameters by using RF, we also obtained good predicted results. Except for Zn^2+^, the results of GBM^(2)^ were better than those of RF algorithm. For Cu^2+^, Fe^2+^, Co^2+^, Na^+^, and K^+^ ligands, the S_n_ and MCC values were at least 26.18% and 0.259 higher in the GBM algorithm. For Fe^3+^ and Mn^2+^ ligands, the S_n_ and MCC values were at least 17.5% and 0.214 higher, respectively.

**FIGURE 8:**
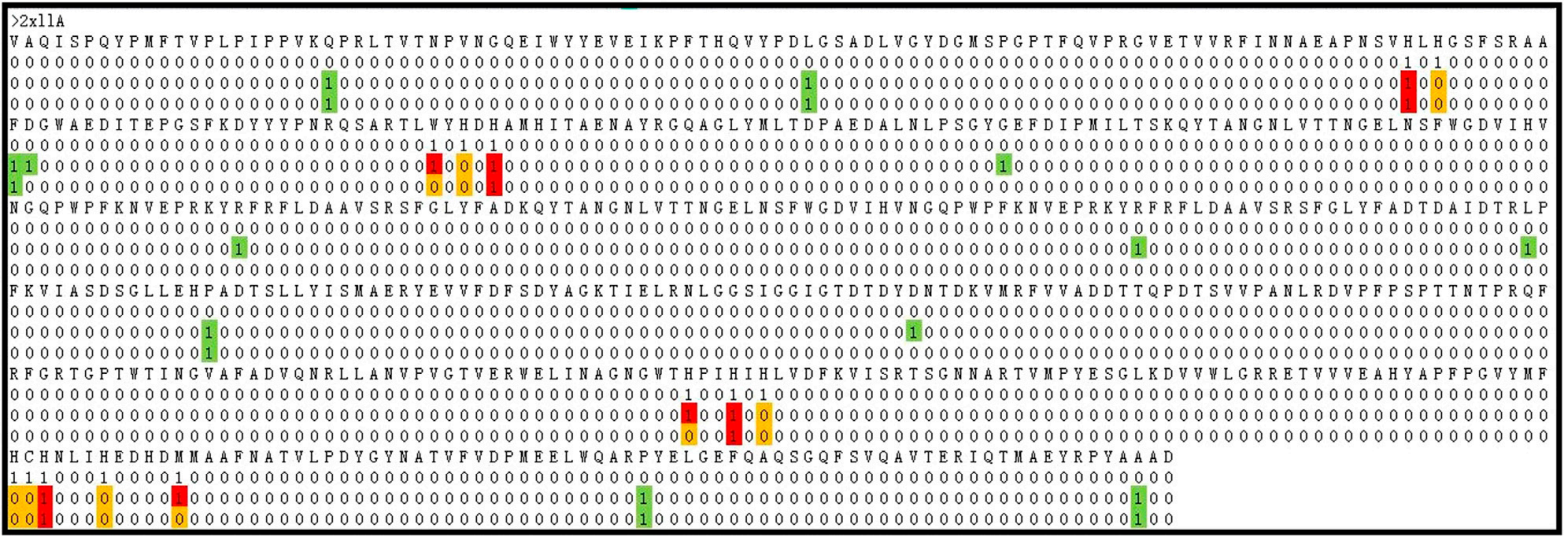
The comparison of identification results. Note: The first row is the protein sequence, the second row is the experimental results, the third row is the optimal predicted results, and the fourth row is the predicted results using the basic parameters. “0” is the non-binding residue, “1” is the binding residue. The red ones indicate TP. The white ones indicate TN. The yellow ones indicate FN. The green ones indicate FP.

In the field of predicting metal ion ligand-binding residues, Hu et al. (2016a) proposed several predicted methods and obtained well-predicted results. At present, the Ionseq is a method with better predicted results on the unbalanced datasets. Thus, we took a comparison with the method of Ionseq in Table 6. It can be seen that the S_n_ and MCC values of Cu^2+^, Fe^2+^, Mn^2+^, Mg^2+^, and K^+^ ligands were better than those of Ionseq. Due to the fact that the number of binding residues was far less than the number of non-binding residues, it would lead to a high false positive. In order to show the improvement, we took a random protein chain (2 × 11A) bound by Cu^2+^ ligand as an example. Based on the above optimal model, we made a prediction for this protein chain. The predicted results obtained are shown in Figure 8.

By comparing the second and third rows, we obtained that the prediction results of the optimal model (GBM^(2)^) were TP = 7, TN = 509, FP = 6, and FN = 11. By comparing the second and fourth rows, the prediction results of the prediction model with basic feature parameters were TP = 4, TN = 514, FP = 6, and FN = 9. The comparison showed that the prediction results were significantly improved after adding correlation features and propensity factors.

## 5 Conclusion

In this paper, based on the primary sequence information, the amino acid correlation features and binding residue propensity factors were added as feature parameters for the prediction of the metal ion ligand-binding residues. In comparison with previous works, our improved results proved that the features of amino acid correlation information and propensity factor information were beneficial to the identification of the metal ion ligand-binding residues. With the optimized parameters, the results of GBM were better than those of RF on the same parameters. Therefore, we believe that our proposed method was a valuable tool to identify metal ion ligand-binding residues.

## Data Availability

The datasets presented in this study can be found in online repositories. The names of the repository/repositories and accession number(s) can be found below: BioLip. (http://zhanglab.ccmb.med.umich.edu/BioLiP/) The key data sets of our work were available in the Supplementary File.
