# Supplementary material for: Recognition of Metal Ion Ligand-Binding Residues by Adding Correlation Features and Propensity Factors
Source: Front Genet. 2022 Jan 4;12:793800. doi: 10.3389/fgene.2021.793800 (PMC8764267; doi:10.3389/fgene.2021.793800)
Supplement: Supplementary file 1 [file Table1.docx]

| Zn2+ | Cu2+ | Fe2+ | Fe3+ | Co2+ | Mn2+ | Ca2+ | Mg2+ | Na+ | k+ |
| --- | --- | --- | --- | --- | --- | --- | --- | --- | --- |
| 2bcoA | 4dyzA | 1unbA | 1gvgA | 3i11A | 2ocgA | 1g9uA | 3cmeT | 1m4yB | 1a49H |
| 3rpcD | 1id2A | 3e1qI | 2rg4B | 2vc7B | 1htoC | 1ayoB | 3zvlA | 3zk1T | 3c33B |
| 4jivD | 3fboA | 1yuzB | 2ohiG | 3thhA | 3ze0D | 1dctA | 2hx1C | 1jz1P | 1hpmA |
| 3qzcB | 3bktA | 2gcuC | 2w8sC | 1t31A | 3ldsA | 2c4dA | 2hv6A | 2bmiB | 1d7uA |
| 4ie6A | 3ppsB | 6prcM | 4aq6D | 4n7qA | 4eweA | 1mpxC | 1x8bA | 4p33A | 1m5hE |
| 1hi9E | 3epvB | 2zo9B | 4hbhL | 3iuhC | 2if8A | 2wyfD | 3tiqB | 3iwjB | 2wmeC |
| 3ebgA | 4alrA | 4gulA | 2csgA | 1v29A | 4h0dA | 4hroB | 3doeA | 1a5uF | 2fcaA |
| 1lr5D | 3gyrE | 1w06A | 1ykpH | 2xtsC | 2cixA | 1upsA | 2y87A | 1tw8C | 4h5jB |
| 1wppA | 1rk5A | 4f2zA | 1k70A | 2xinB | 3fxlA | 2c5uB | 2c5uB | 3goxA | 1jjwC |
| 1wgzA | 1ibyC | 1dv6L | 4qqwA | 2xjmD | 4k28B | 3l8yA | 2xj9A | 1glhA | 2qv6A |
| 3eyvA | 1pzsA | 3ak9A | 3ldbA | 2b3kA | 4m0vC | 4q51A | 1rlmD | 3bk8A | 1r64A |
| 3bywC | 3n7eA | 1oaoB | 4fm4C | 3schA | 8icpA | 3h6gB | 3qxcA | 4eiyA | 2bkpA |
| 2vnfC | 1m57B | 4oj8C | 2gbwE | 2hn1A | 4ls9B | 2c3oA | 3mtcA | 3c32A | 4jayC |
| 3it7B | 3tyhG | 1mpyA | 2iuwA | 2ps9B | 4nj4A | 3p85A | 2c3oA | 2dsnA | 2oecA |
| 3kdkA | 4oy7A | 3pl1A | 4k19C | 1l8xB | 1kgpC | 4fsoA | 1ys7B | 4jt4B | 1gkzA |
| 2rjbB | 3g5wB | 3wsiA | 2jblM | 4gjyA | 4ifzA | 2pqxA | 4g61A | 2bl2E | 3hw9B |
| 2v2aA | 1jerA | 3vmhC | 3l0pA | 3rmuD | 3llmA | 4hxbL | 2dgnA | 1jouF | 3fd5B |
| 3qsuD | 3tasB | 4hz1B | 4m6xA | 2fuaA | 2zadB | 2clpC | 4hubL | 1rv8D | 2fxiA |
| 1llmC | 3nbbB | 1oqbF | 3dhiA | 3n5fA | 3lopA | 3flrA | 1we3A | 1o8uE | 1jdbF |
| 1q08A | 3aasA | 3aqjA | 1nmoF | 1tu4C | 1gq2N | 4m6xA | 3tavB | 1ba1A | 1krjA |
| 3fnsA | 1ksiB | 1cjxA | 3bvlF | 1wmpB | 4ewvA | 2jg9D | 1tt4A | 2wuvA | 1ax4D |
| 1dzuP | 2y9xB | 4il6D | 3hh8A | 1rxtC | 4ccnB | 3qm2B | 4okzD | 1e3zA | 2ibwC |
| 4fwjA | 3k7rE | 1jr7A | 3d19A | 1b59A | 2hxgC | 3htlX | 2fkzD | 4ntxA | 2j41D |
| 3v24N | 3de8B | 1nf4I | 3eqeA | 1stgA | 2o1eA | 3cu2B | 3fqxA | 3ndhA | 1ud6A |
| 2z8fA | 3e6zX | 3lfmA | 4kkzD | 1cobB | 4oa9A | 4jjjA | 3dkcA | 2olnA | 1jdbK |
| 4uq7B | 1odbE | 3bz2A | 1w2aX | 4g3qA | 1uvmA | 1ul3B | 4ay7B | 2jlnA | 2whvA |
| 1su1B | 3mztB | 3k9zA | 1z02A | 3tk4A | 2b2kB | 3ge4I | 3i0oA | 3zuyA | 2pa2A |
| 4griB | 2wq8A | 4b2oA | 3nnfA | 1rrkA | 3kquE | 3bcfA | 4ifzA | 2yxuB | 3zd9A |
| 3g7lA | 1asqB | 3jskN | 2gm6A | 3e3hA | 3s23A | 1kkmB | 2yniA | 3me4A | 1fpiA |
| 1qu2A | 3mn0A | 2xgfC | 2jb4A | 4cv7A | 1m0dC | 1rtp2 | 3lopA | 4eelA | 3fgoB |
| 4n7sC | 4hd6A | 4kwjA | 3fpvD | 1t5kA | 3pifC | 1ys6B | 1bwvC | 1ag9B | 4it1C |
| 1fbxB | 4hcfB | 1rsrB | 1j3qA | 1fsqB | 4fliA | 1ydnC | 1h74B | 3pncA | 1dtwB |
| 2anpA | 3t6qA | 3ayzA | 4rxnA | 2claA | 3orkA | 3wd9B | 4dfdA | 3b9bA | 2a0qD |
| 4oh1A | 4lsyB | 1smwA | 3c25A | 4q0uA | 4e2qA | 4h2iA | 3nemB | 3lg1A | 3ss8B |
| 1mftB | 2fouA | 3wr9B | 3qgkI | 3v9uD | 1xldA | 2fkcA | 2hkjA | 1lokA | 1wcfA |
| 1tnbJ | 3zjaA | 4nm6A | 1dmhA | 2icvA | 1txoB | 3w7uA | 3f79E | 3paqA | 1dioA |
| 3tw6D | 2ztkA | 3gzyA | 1mtyE | 2zu9A | 3ol8A | 2dbxB | 1w78A | 3r0xA | 1dtwA |
| 3balB | 4e4zA | 4ou9A | 3qhbB | 3mf3B | 1g0iA | 1g8hB | 2q0yA | 1ksuB | 1ttqB |
| 3bofA | 1cc3B | 1gp6A | 3fvbA | 1vlxD | 4gikB | 2ejnA | 3mg8L | 3b6pA | 3crlB |
| 1ocrS | 2xmkB | 2wboA | 2vunD | 2uzpC | 3t7mA | 4abtB | 4ffoA | 3fd6B | 1gzgB |
| 2jd8K | 1h1iB | 4kbzA | 2q4aB | 6celA | 3ufxB | 4g1eA | 4qjlA | 2gezC | 4a0mA |
| 3mhsC | 1s4cB | 3mguA | 3wfdB | 1uv5A | 3csbA | 2ozbE | 4fwiB | 3ifvC | 1nhiA |
| 3r4xA | 3awuB | 3vshD | 2fjcB | 3g5pA | 1de9A | 3ovsA | 2gjkA | 4jb3A | 3umoB |
| 3v2dY | 4oakA | 1wa6X | 4d8gB | 259lA | 4qkfA | 2bs9E | 1g8hB | 1i40A | 2xptA |
| 3ohcD | 2b7jC | 2fdjA | 4ergA | 1c4gA | 1bxrC | 1ti2A | 3s89C | 1b57A | 4twkA |
| 4koyA | 2fk1A | 4o6jA | 3fm6A | 4aq4A | 1de6A | 4fjiA | 1vx9P | 4ekfA | 1w22A |
| 4kfuA | 4mntA | 3fg3A | 3o1vA | 2fqoA | 4eemA | 3ai7G | 3v23Q | 1nhjA | 2a6vA |
| 1i50C | 3higA | 2z90A | 1xm8A | 2amxA | 3hq1B | 2gftB | 1qu2A | 4bvnA | 1gupA |
| 1cz0A | 2uwfA | 1rrlB | 3ivdA | 2zc1A | 3ea3B | 1xxmD | 3cmwA | 2iwfB | 2b5wA |
| 2dq4A | 2yc3A | 3nl1A | 2uyuA | 2bo7J | 3ctzA | 3ayfA | 1z72B | 2q6hA | 3s3xA |
| 1tjlF | 2xz4A | 2fctA | 2ydeA | 1jn1A | 3w42A | 4ln6G | 1wpvB | 3dh4D | 1me7A |
| 2v86B | 2c9qA | 4h3eA | 4mcwB | 1r0hA | 3telA | 4linF | 4d09B | 3ntuA | 2zxeA |
| 4c3iI | 3od3A | 1mmkA | 1rsvB | 1k1eE | 3ivdA | 4o6nB | 1ig5A | 2ymeI | 4m3pB |
| 3sxkA | 4h7lB | 3ussB | 3aalA | 2bdiI | 1gv3B | 2ccmA | 4kxwA | 3nvdA |  |
| 1xllA | 4eirB | 1h5zA | 3ufkA | 1of8B | 1i0bA | 2re1A | 1aqfH | 2iajA |  |
| 4lc5A | 2xjnK | 3r60A | 4iwjH | 2ew5A | 2bo8E | 3s4fA | 2fcoA | 2j5wA |  |
| 1ychA | 3iudC | 3n9mA | 1o2dB | 3ut1A | 1h48D | 1g8kG | 4qcr2 | 4g8tB |  |
| 4w6zD | 1t16B | 1os7A | 2vzbA | 3nzhH | 3gg7A | 1g20B | 3fkqA | 2x2eD |  |
| 1jmuH | 2j5wA | 4m51A | 3t81A | 3ojkD | 3vylB | 3salB | 3ct7E | 3dyqB |  |
| 3amjA | 3wa2X | 4naoA | 2biwB | 1rv8D | 3b59D | 2h2uB | 4b3tN | 1suzA |  |
| 3l8eA | 2xlaC | 2zi8A | 1auiA | 1gwmA | 1s3nB | 3lmwA | 3tw6D | 2hzlB |  |
| 2oi0A | 2voxA | 1fz0A | 1aorB | 2os3A | 1b8aB | 3kkfA | 3cq3D | 2fmqA |  |
| 4ngnA | 2zcvA | 4ghcC | 3bv6E | 1vz0D | 4edkA | 3kzpB | 3q45A | 3syaA |  |
| 4mbsB | 4flmB | 2yu2A | 1tjoA | 2z06D | 3fcmB | 1l6rB | 4hnzJ | 1ka0A |  |
| 2g3fA | 3gdcA | 1sp8D | 4jo0A | 3l0sA | 3n3cA | 2i4cA | 3ahcA | 2rhjA |  |
| 4k6mB | 2iwkB | 2yc0A | 4f9jA | 4jh5A | 1w2zB | 3q6dA | 4kj53 | 3ondB |  |
| 3ld0R | 2e46A | 1h7aA | 2pybA | 3zr9A | 1kflA | 2qpuA | 1m3uI | 4kcaB |  |
| 2xevB | 4o65A | 3ovpB | 2gp3A | 4askA | 1bfrB | 1yleA | 3qfvB | 2zhjA |  |
| 4gnxC | 3b1jA | 2ehzA | 1z6oW | 1rmqA | 2wodB | 1e54A | 1m1bA | 1mwvB |  |
| 1dq3A | 1thoA | 1rrmA | 3gteA | 3r4qA | 3m5qA | 4bcuA | 3oevK | 1lkbA |  |
| 2c20E | 1r5aA | 2pt2A | 4ewaA | 3m4zA | 3nqwA | 4b0pA | 2q5zB | 1bgpA |  |
| 4fvkB | 1bugA | 4j1xC | 2yxoB | 1fofA | 4ipiA | 3hz2A | 4klnA | 1h80A |  |
| 4dglB | 2oxiA | 3veoA | 2ji3C | 3gobC | 1oi8A | 3kqaD | 1amuA | 1me7A |  |
| 4ih3C | 4phzI | 2pxjA | 2xv1A | 2xdmA | 1wrnB | 1uf3G | 3jz0B | 3nmbA |  |
| 3ea1A | 2fu7B | 4hr4A | 4ha0B | 4m5cA | 1fggA | 2vvfC | 1svwA | 4matA |  |
| 2zh0F | 1ov8B | 2vw8A | 3fmrA | 1rqeA | 4ewtC | 3v7zA | 4c7yA | 4gfiC |  |
| 4e7hA | 3umkA | 2r1lA | 1kmpA | 4jbhA | 4k1cA | 2fi4I | 2pk0A | 1gzgA |  |
| 4k90A | 4hvoA | 4g33A | 3lmcA | 4ft8A | 3ckqA | 3m83B | 1jd2G | 2j9yB |  |
| 4dwxA | 1gy2A | 1e5sA | 1nx8B | 3dwcB | 2yb1A | 2q69A | 1ovdA |  |  |
| 3jygE | 2jlpD | 3mz6A | 1shrB | 1r8kA | 3n9bB | 3oorA | 3ey9B |  |  |
| 3aygA | 1a8vB | 2ivpA | 2z36B | 3d03F | 1jqcB | 3oheB | 1uutB |  |  |
| 2f5qA | 1aqpA | 2ji2A | 3zpoA | 4gprA | 1woiF | 3edkB | 2whiE |  |  |
| 2jlpD | 4j3rA | 2bq8X | 4qlwD | 2j3zB | 1peyB | 3ab9A | 2i5rB |  |  |
| 1kapP | 1lcfA | 2a1xA | 1xvxA | 2xvzA | 2uybA | 3q09B | 3h87A |  |  |
| 1z83A | 1gmwA | 3obzA | 1ltvA | 1hgwA | 2z86C | 2rhpA | 2r8eH |  |  |
| 4l99B | 1v6pB | 4bmqA | 4kvqA | 3i8vA | 4m8dD | 4f5yB | 3ejaA |  |  |
| 3fp5A | 2gimC | 1y0zB | 2puzA | 2parB | 4e1dA | 2ot4A | 2vzbA |  |  |
| 3mpvB | 1zm5A | 4qddA | 4mlmA | 2xphB | 2ongB | 2e6uX | 3vnpB |  |  |
| 2gc3A | 4lejA | 2qh1B | 2c6rA | 1m38B | 3idbA | 1o9iA | 4qcpD |  |  |
| 1dwfM | 4n3uA | 1w69A | 4b8yA | 4jz2A | 1yd3A | 3w9tF | 2ihvD |  |  |
| 3ju2A | 4phzC | 1zt5A | 4olsD | 4jkaB | 2qjtB | 3q13A | 3pdeD |  |  |
| 3dynA | 3zx1A | 1ey2A | 1lnbE | 2c79A | 4e7iA | 4jdzB | 2rcyA |  |  |
| 2zc2B | 3pgbA |  | 3maoA | 4eraA | 4lomA | 3sjsA | 4c0lA |  |  |
| 2hf1A | 1s1qA |  | 1sumB | 1bk5B | 1wao3 | 1dx5K | 2vbvB |  |  |
| 3bknL | 1xmeA |  | 1y4tA | 3alqS | 3bamA | 3m5qA | 1ne9A |  |  |
| 1k0wD | 3sb8A |  | 4mhuA | 4i2gA | 1ystL | 1j0nA | 4obbA |  |  |
| 3l7tB | 1zxiB |  | 3qc3A | 2yyeB | 3bsoA | 3avwA | 3w9tF |  |  |
| 3fidB | 2yevD |  | 3ks5B | 3bd5B | 1nb7A | 4ngnA | 3vatA |  |  |
| 1hqaA | 1sddB |  | 4ku0D | 3ttbA | 1cnzB | 3ip7A | 2hw5D |  |  |
| 3oaxA | 1ukuA |  | 3cpxB | 3r61A | 1d8hB | 4jucB | 4as5B |  |  |
| 3wl3A | 4jfnA |  | 3fvzA | 2wyrH | 4jh9A | 1imeA | 2zrzA |  |  |
| 1rjqA | 3ziyA |  | 4nd8D | 3h9aB | 1rqqA | 1qi5A | 1kd14 |  |  |
| 1ld3A | 3u52A |  | 4nubA | 3kolA | 4n7tA | 1i22D | 1t5jA |  |  |
| 1xruA | 3sesA |  | 2amuA | 4litA | 2vs8F | 3u48B | 1gljY |  |  |
| 2vxxC | 1iaaA |  | 4f0lB | 1lnaE | 2wymA | 3fkhC | 1cmcA |  |  |
| 4ov9A | 1oacB |  | 3u9mE | 1qxyA | 3ck2A | 2voxA | 3ig5A |  |  |
| 4je7A | 3zudA |  | 3li2B | 4metA | 4ic1D | 4ovdA | 1urbA |  |  |
| 2oh3A | 4kavA |  | 3fe5A | 4jkyB | 2p0nA | 1j9kB | 2p06B |  |  |
| 2fgyA | 3e0iA |  | 3r2uA | 3w45B | 3dw9A | 2yb7B | 4iokB |  |  |
| 2waeA | 4nunA |  | 2bi4B | 2gruA | 2enxA | 3u4jA | 4f6tB |  |  |
| 1t8hA | 3lzqB |  | 1oquC | 1xmhB | 3q23A | 4fyag | 1q19D |  |  |
| 2pmsD | 3w6wA |  | 4e4hB | 4nncA | 4mr0A | 4k1cA | 3f1eE |  |  |
| 3msuB | 2aqsA |  | 3zk4B | 3pniB | 4irqC | 3d4gG | 3a7rA |  |  |
| 2bteD | 4b61B |  | 3bxvA | 2y8uB | 3ujqA | 3gdcA | 4qcrW |  |  |
| 4icsA | 3tu6A |  | 2yeqA | 3vxmE | 3frxB | 3cqoC | 1ryaB |  |  |
| 1it8A | 3hhsA |  | 3cf4A | 3lmmD | 3ilmC | 1cp9A | 3u4mA |  |  |
| 2psrA | 1ekjG |  | 4huzA | 1y6vA | 2xi5A | 3nvnB | 1n0hA |  |  |
| 4nj5A |  |  | 3l87A | 1a0eD | 2phnB | 4pqiA | 2gx2I |  |  |
| 4qf3A |  |  | 3s6bA | 3mgpD | 1ucgB | 1ht9A | 1v14A |  |  |
| 1r44E |  |  | 3tlkA | 3pjlA | 4pxbB | 1j12D | 3es7B |  |  |
| 1jk0A |  |  | 2clbC | 3oceA | 2qb5A | 4lmfD | 3fefC |  |  |
| 1q3kC |  |  | 2itbA | 2ddsA | 2g38B | 4h15B | 1wpgA |  |  |
| 4g23A |  |  | 4b28A | 3m5aA | 3pfpA | 4n3oB | 1efkC |  |  |
| 3fkgA |  |  | 1warA | 3sxxA | 2ga2A | 1k1xB | 4bvrB |  |  |
| 3m2uA |  |  | 2yjkH | 2ehdB | 4pssA | 3iqeD | 1iw7O |  |  |
| 3esjA |  |  | 2ogiB | 4afoB | 3f05A | 4k90A | 3ld0U |  |  |
| 3q7jB |  |  | 4qdcA | 3hdiA | 4j6oA | 2vmeD | 1fiuC |  |  |
| 2zxcA |  |  | 1nf6F | 3s5hA | 3p2uB | 3ossD | 4hb4A |  |  |
| 3kjiA |  |  | 3bxdA | 2f7nA | 2wc9A | 2oaaB | 3kwsB |  |  |
| 2anuF |  |  | 3wsfA | 3nvlB | 2f5cA | 2whkA | 4nx8A |  |  |
| 4qd14 |  |  | 2w16B | 3ggdA | 3hmkB | 4kppA | 3knhO |  |  |
| 1e67A |  |  | 2phmA | 2f0aB | 2jecD | 3ehjB | 3eywA |  |  |
| 3tgnB |  |  | 1y07C | 1wn1B | 3f2cA | 1xcbA | 3zvhB |  |  |
| 2drpD |  |  | 1veiA | 1gqjA | 1n38A | 4jrfA | 3a4wB |  |  |
| 1khoA |  |  | 2b5hA | 1jymA | 3rvaA | 1mr8B | 4mzuB |  |  |
| 3un6A |  |  | 3h9aA | 2dfiA | 2bffA | 2b30B | 2a69C |  |  |
| 3csqD |  |  | 1y56A | 4ekdA | 1jlkA | 4nsdA | 3bhtB |  |  |
| 1q95I |  |  | 1yuzB | 3gqaA | 2o5aA | 2c9mA | 3cbtA |  |  |
| 3didA |  |  | 1y67D | 2effA | 1xmsA | 1xmfC | 4q04A |  |  |
| 2g64A |  |  | 3dbyT | 2y3bA | 1e24A | 1kapP | 3h7vA |  |  |
| 3ltdA |  |  | 3oghB | 4iwwB | 4gaeA | 1qdbA | 3nm7A |  |  |
| 2i14C |  |  | 2hk6A | 1fa6B | 3v1rA | 1m1jB | 3hdgA |  |  |
| 4hi8B |  |  | 3vsiB | 3p0fA | 4e19A | 3gwzD | 3ohzF |  |  |
| 3qu6A |  |  | 3qy8A | 3tghA | 3cm5A | 2q17E | 4nkbA |  |  |
| 2b5lD |  |  | 2j2fA | 1h0nA | 3fyoD | 2wqdA | 2vn2A |  |  |
| 4p10A |  |  | 3vcpA | 4eroA | 3h0rK | 3ubhA | 1dakA |  |  |
| 2bq8X |  |  | 3o0fA | 3nykA | 2z7bA | 1a25A | 3knhF |  |  |
| 2jigA |  |  | 3vtoB | 2gliA | 4ogcA | 3njvA | 1nj1A |  |  |
| 4h9kA |  |  | 2q0jA | 3mcrA | 3r3lB | 3akbA | 4hxgF |  |  |
| 3b6pC |  |  | 2innA | 1iabA | 2ztiA | 1n7uA | 2y4mB |  |  |
| 1oaoC |  |  | 3qy7A | 1ukwB | 1vj7A | 4bq4A | 4dtfA |  |  |
| 4ehcA |  |  | 1no3A | 1qreA | 1a76A | 2gp7A | 4bpwA |  |  |
| 4dlmA |  |  | 2zziB | 1scsA | 1dckB | 3pvoD | 4qcz3 |  |  |
| 3ad9D |  |  | 2gyqA | 3ka9A | 2x7jB | 4n6fB | 2quiA |  |  |
| 3bvvA |  |  | 1rcwB | 2ywrA | 1xhvA | 3f1vB | 4qcnH |  |  |
| 2olmA |  |  | 3qxbA | 3tw3A | 1fi2A | 3bjeA | 1q6oB |  |  |
| 1hz5B |  |  | 1bszA | 3tr3B | 1dahA | 3fawA | 3c4qB |  |  |
| 4a48B |  |  | 2h0vA | 2vosA | 1i5aA | 1hkbB | 3lasB |  |  |
| 2gbxD |  |  | 2vhlB | 2xdbA | 4fbkA | 4awnA | 3o5tA |  |  |
| 2byoA |  |  | 3vthA | 2j4jF | 1u3eM | 4nczC | 1qhyA |  |  |
| 1qq9A |  |  | 2xmoA | 3khcB | 4bzcC | 2ykkA | 4eo7A |  |  |
| 4o62B |  |  | 1zgnB | 4magA | 2qgiA | 3ldyA | 3vayA |  |  |
| 1piwA |  |  | 1t71A | 4b5wA | 3lzqB | 2aerL | 1v5fA |  |  |
| 3v8eF |  |  | 1cojA | 3ta5A | 1twfA | 2c26A | 2qojZ |  |  |
| 3gdfB |  |  | 1vljB | 2qb7A | 4lfiB | 4nuzA | 3na8C |  |  |
| 2bibA |  |  | 4aiqA | 2prqA | 2vqrA | 3fzxA | 4qcrN |  |  |
| 2einC |  |  | 2p4zB | 4nuiA | 1ef2A | 2yfsA | 2yfnA |  |  |
| 4o23A |  |  | 2i9uA | 4cv4A | 2a9iA | 4h0nC | 4qbyI |  |  |
| 3ndiA |  |  | 3e74B | 1r6xA | 2pyjB | 4oy7G | 2g8hA |  |  |
| 2g2nD |  |  | 1tmxA | 2oi6B | 2hbkA | 3lagA | 3ed6A |  |  |
| 3lggA |  |  | 3pu8B | 4iynB | 1qprF | 4celA | 1cw0A |  |  |
| 3cewA |  |  | 3d3lA | 4n8mA | 1fa0B | 3co6C | 3c22D |  |  |
| 1y9aC |  |  | 4ne0A | 3rgtB | 2v8uB | 2iwvC | 2a9fA |  |  |
| 3g1pB |  |  | 3kt4A | 2f7vA | 3dysB | 3psrA | 2e67B |  |  |
| 2nytB |  |  | 1frfL | 2q6qB | 3t9qB | 4bz4B | 2x3jA |  |  |
| 1e7dB |  |  | 2wl9A | 3ivuB | 2mnrA | 2iwaA | 1xmqJ |  |  |
| 4dzhA |  |  | 1q0oB | 3h2wA | 1o99A | 2pltA | 3bwlB |  |  |
| 1gl4A |  |  | 3qr7B | 2opcA | 2amhA | 1kuhA | 1p5zB |  |  |
| 4mhqA |  |  | 4jydA | 3uc3A | 1ipsB | 2g42B | 2oiwC |  |  |
| 4p6rA |  |  | 2c2fA | 3si8A | 3r7pA | 1uppE | 4ltzA |  |  |
| 3r0dA |  |  | 3wpmA | 2jf5B | 2palA | 1z6oC | 4qcr8 |  |  |
| 3ib2A |  |  | 2fiyA | 3krgA | 2iocB | 2iicB | 2aerL |  |  |
| 4on1A |  |  | 3qfmA | 4njqB | 2b51A | 1u2vA | 4tq4D |  |  |
| 2r2dE |  |  | 3bwwA | 1xrcA | 3ojnB | 1sl6B | 1nlqE |  |  |
| 4brrD |  |  | 1xsmA | 1nr5B | 2d7iA | 4hwvA | 2bhdA |  |  |
| 4r1mA |  |  | 3htnB | 1zjcA | 4gpuA | 1ka1A | 4fvuA |  |  |
| 3ewdA |  |  | 3lm4D | 3cunB | 1zp9A | 3tquB | 1znoB |  |  |
| 4f56B |  |  | 3v7pA | 3mz7A | 3lf1B | 2vnvE | 2pmqB |  |  |
| 3rgbK |  |  | 2yivX | 3qq7A | 3c5mB | 1a0sR | 4dpvZ |  |  |
| 1mbxA |  |  | 3ktcB | 3s8kA | 3vnlA | 1suiC | 3n2nD |  |  |
| 2o6dA |  |  | 2iw4B | 4as7A | 2id0B | 2zyhB | 4ev0A |  |  |
| 4kf9A |  |  | 1wraB | 1fx7A | 1jfzB | 4ak7A | 3ocvA |  |  |
| 3nqzB |  |  | 4j5iB | 3isqA | 3qfkA | 4ko1M | 4ariA |  |  |
| 2q02D |  |  | 2rdnA | 2r5vA | 1c39B | 3wctB | 3k9fC |  |  |
| 3pihA |  |  | 3perA |  | 4bifB | 1kdmA | 1h56A |  |  |
| 2gwnA |  |  | 3saoB |  | 4c8dA | 2bamA | 2yevD |  |  |
| 2vrsC |  |  | 3ib7A |  | 2am3A | 2iewA | 2yvmA |  |  |
| 2oajA |  |  | 4ac8A |  | 2jlbA | 4a15A | 4dkwB |  |  |
| 1yg9A |  |  | 3zk3A |  | 2jcdA | 1yi7D | 2a87A |  |  |
| 3l4kA |  |  | 3hc1A |  | 1vj2A | 4lxoB | 1sojD |  |  |
| 4g6uA |  |  | 3hq0A |  | 3i3qB | 4fgcB | 3uqeA |  |  |
| 2cihA |  |  | 4fagA |  | 3gmeA | 1g0hB | 3pdtA |  |  |
| 4lkaA |  |  | 2bjjX |  | 1eccB | 3gqfB | 1iruU |  |  |
| 3nnqB |  |  | 2pq7A |  | 4migC | 1c9uB | 1q9sA |  |  |
| 3h3eA |  |  | 2wluA |  | 1zu0A | 3f6yA | 5xiaA |  |  |
| 2feaA |  |  | 4cybJ |  | 1lncE | 1jb0L | 1ka1A |  |  |
| 4k88A |  |  | 3tmbB |  | 3v0qA | 2y6jA | 1fjgL |  |  |
| 2zo4A |  |  | 3rf7A |  | 3bg5A | 4afxA | 1it8A |  |  |
| 4afsA |  |  | 1sqdA |  | 2pnyA | 3dbkA | 3d6wA |  |  |
| 2i0mA |  |  | 1gp5A |  | 1up6A | 2w66B | 4ip2C |  |  |
| 2ek9A |  |  | 1gupA |  | 1za0A | 1blxA | 3koaA |  |  |
| 3mfqA |  |  | 3pqiA |  | 3azmE | 3bwxA | 1mowA |  |  |
| 4eg2A |  |  | 3vv9B |  | 1g5bB | 1t1eA | 4gp7A |  |  |
| 2zneA |  |  | 3nteA |  | 3g3rB | 2d00C | 3l23A |  |  |
| 3hcjB |  |  | 3omiC |  | 1jlmA | 1gttD | 4cglA |  |  |
| 1sedC |  |  | 3dcpA |  | 3n10A | 4h7jA | 4as2B |  |  |
| 1zswA |  |  | 1vrbD |  | 1ro6A | 3tkkC | 3akmD |  |  |
| 3csvA |  |  |  |  | 4hcxB | 2gsyG | 4pyoB |  |  |
| 4avxA |  |  |  |  | 2py7X | 3rhtD | 3au9A |  |  |
| 4ct0B |  |  |  |  | 2xmoA | 2wp4B | 2npiB |  |  |
| 4gizD |  |  |  |  | 4ez1C | 3q9mC | 2pgoB |  |  |
| 3q05B |  |  |  |  | 1e6aA | 1u5qA | 3etjB |  |  |
| 3ttsD |  |  |  |  | 2oknB | 1yaxA | 2o1oA |  |  |
| 1q3bA |  |  |  |  | 1p3dA | 3fd3A | 2icpA |  |  |
| 2nvvD |  |  |  |  | 3repA | 3l1vA | 2yi9C |  |  |
| 1ibqA |  |  |  |  | 4kpyA | 4g1eB | 1d2iA |  |  |
| 2qq4H |  |  |  |  | 4iw3J | 3b7yA | 4mpoB |  |  |
| 2zwiB |  |  |  |  | 2wdeA | 2i8tB | 3cmeB |  |  |
| 3h8fF |  |  |  |  | 3s5aA | 1tkcA | 1jylC |  |  |
| 2bx2L |  |  |  |  | 1w4aC | 2w87A | 4qcr1 |  |  |
| 3ifeA |  |  |  |  | 1a0dC | 1m57G | 1svmC |  |  |
| 2rhsC |  |  |  |  | 2faqB | 1vldX | 4nb4F |  |  |
| 2c3aB |  |  |  |  | 2vbeA | 3a6zC | 2px0H |  |  |
| 1ndzA |  |  |  |  | 3rmjB | 4kn9T | 3mf4B |  |  |
| 3q7aB |  |  |  |  | 1zaoA | 4sgbE | 4nvvR |  |  |
| 3kycB |  |  |  |  | 3fijH | 1tlqA | 1n32D |  |  |
| 4mj7A |  |  |  |  | 3h8fF | 3ef2D | 2uu7I |  |  |
| 1h7rA |  |  |  |  | 4dm0A | 2w22A | 2vjlA |  |  |
| 3cneD |  |  |  |  | 2e6cA | 1lw5D | 2zxcA |  |  |
| 4jnjA |  |  |  |  | 1xmfB | 3mcyC | 4if4D |  |  |
| 1vpyA |  |  |  |  | 4pcaA | 2vn7A | 1b8cA |  |  |
| 3tkkA |  |  |  |  | 3a4kD | 3wu2c | 1gtvA |  |  |
| 3w0fA |  |  |  |  | 4ln7A | 4adgC | 2zanA |  |  |
| 1p6oB |  |  |  |  | 2w5qA | 3qfrA | 2xhvB |  |  |
| 3ovgA |  |  |  |  | 3nqbA | 4ar9B | 3t5pL |  |  |
| 2h1iA |  |  |  |  | 3gbrB | 3cniA | 3wgvA |  |  |
| 4nu7C |  |  |  |  | 3g0zA | 1t6cA | 2y4aD |  |  |
| 3ls1B |  |  |  |  | 2q0mX | 3ur3C | 1d9dA |  |  |
| 4onxE |  |  |  |  | 3pzlB | 1gxrA | 4h0iA |  |  |
| 1u0bB |  |  |  |  | 3qszA | 3lzkC | 2fbxA |  |  |
| 4l9mA |  |  |  |  | 1n1pA | 5stdA | 2as8B |  |  |
| 4k0dB |  |  |  |  | 3gfzB | 2wysB | 2bphB |  |  |
| 3ngjD |  |  |  |  | 3u95B | 3voaA | 4nvvG |  |  |
| 3lx3A |  |  |  |  | 1cw1A | 2e7fA | 4gzkA |  |  |
| 4fuoA |  |  |  |  | 2yf3D | 1kwhA | 1ow2B |  |  |
| 3dowA |  |  |  |  | 3d2oA | 3vl7A | 2b1qA |  |  |
| 2r8qA |  |  |  |  | 4i2cA | 1hm9A | 1miwB |  |  |
| 3g27A |  |  |  |  | 3qh8A | 2hesX | 2ad5B |  |  |
| 3e2dA |  |  |  |  | 1sx5A | 4asmB | 1e1zP |  |  |
| 2vwhA |  |  |  |  | 3ib7A | 3qypB | 3nvsA |  |  |
| 3cghA |  |  |  |  | 1o4tB | 1qlbD | 2yxhB |  |  |
| 3axsA |  |  |  |  | 1ss9A | 2vqrA | 3e48A |  |  |
| 1zy7A |  |  |  |  | 3psnA | 1lomA | 3rimC |  |  |
| 2cksB |  |  |  |  | 1vsfA | 3zq4A | 3v2uD |  |  |
| 4fw6C |  |  |  |  | 2qvwA | 1uv4A | 2hwgA |  |  |
| 2axrA |  |  |  |  | 4l78A | 1qq9A | 4bkmA |  |  |
| 1dv6H |  |  |  |  | 2alyB | 3q4gA | 1fx4A |  |  |
| 1xcrB |  |  |  |  | 1i19B | 4j7mA | 2ji6B |  |  |
| 4dt2B |  |  |  |  | 2iieA | 2porA | 2g0wB |  |  |
| 4ixnA |  |  |  |  | 4nfwF | 2y5pA | 3fvyA |  |  |
| 1i0dA |  |  |  |  | 1imdB | 3rmkA | 2j1nB |  |  |
| 4b87A |  |  |  |  | 2wjfA | 2je9D | 3mesB |  |  |
| 4gelB |  |  |  |  | 1qpsA | 2o6hA | 4e6mA |  |  |
| 2ux1G |  |  |  |  | 4bulA | 4pibB | 3f1fS |  |  |
| 4ijdB |  |  |  |  | 1jqnA | 2il1A | 2d2fA |  |  |
| 1tafB |  |  |  |  | 3tc3B | 2pooA | 3f61A |  |  |
| 3o14B |  |  |  |  | 1s4pA | 4i72B | 3deqD |  |  |
| 3oj6B |  |  |  |  | 3qb5K | 2bibA | 3r76B |  |  |
| 2osoA |  |  |  |  | 1wsgD | 4b56A | 2x7aI |  |  |
| 4gdfE |  |  |  |  | 3pyzA | 4a9xA | 3sduA |  |  |
| 1kfiB |  |  |  |  | 2jebA | 3qhqA | 2f9rC |  |  |
| 3eiiB |  |  |  |  | 3ki9A | 1r64A | 2q40A |  |  |
| 2fawA |  |  |  |  | 2bvlA | 3c63D | 2amcB |  |  |
| 3v4jA |  |  |  |  | 2pmlX | 3noiB | 3f85A |  |  |
| 3b5qB |  |  |  |  | 4eayC | 3e4qA | 2qvrA |  |  |
| 3mmdA |  |  |  |  | 3thoB | 2ev5A | 2ejwA |  |  |
| 2vl6B |  |  |  |  | 3iduA | 1d0lA | 1ec9D |  |  |
| 3feqJ |  |  |  |  | 4lilA | 4kwuA | 4a48B |  |  |
| 1u10C |  |  |  |  | 4ggfV | 1exzA | 3v5wA |  |  |
| 3hcsB |  |  |  |  | 2zxqA | 4jbeB | 3obkH |  |  |
| 2pliA |  |  |  |  | 3rl3A | 2pmyA | 1pg4B |  |  |
| 2nvqB |  |  |  |  | 1ybuA | 3i57B | 4qczX |  |  |
| 3cg7A |  |  |  |  | 2glfA | 4eqbA | 2zyrA |  |  |
| 3v235 |  |  |  |  | 1mqwA | 3mliD | 2o4gA |  |  |
| 4oy8A |  |  |  |  | 1elsA | 1dykA | 2ag0A |  |  |
| 1m2vB |  |  |  |  | 3mpbB | 1e7dB | 1zotA |  |  |
| 3rqzC |  |  |  |  | 1itwD | 2zktB | 3v8eF |  |  |
| 4bp0A |  |  |  |  | 3zt9A | 3mk1A | 3s1aA |  |  |
| 2e88A |  |  |  |  | 1y63A | 2b9lA | 2dcnB |  |  |
| 2fz6A |  |  |  |  | 3vesA | 1uy4A | 4q3bD |  |  |
| 4pziA |  |  |  |  | 4g2cB | 3n0aA | 3qlzB |  |  |
| 3hpsB |  |  |  |  | 4kirA | 3fozB | 4cogC |  |  |
| 2qeeE |  |  |  |  | 3mxaA | 1r6vA | 2r42A |  |  |
| 1pmiA |  |  |  |  | 2r8dA | 4b7bA | 3qe6B |  |  |
| 1hw7A |  |  |  |  | 2dtiB | 2e1pA | 3mcoA |  |  |
| 1jkeA |  |  |  |  | 1bvaA | 3bs6A | 1y9aC |  |  |
| 2w8sC |  |  |  |  | 1rm0A | 1hdhA | 4hzdA |  |  |
| 4pt5A |  |  |  |  | 2ns6A | 3lp9D | 2xsqA |  |  |
| 4ntlA |  |  |  |  | 3w5wA | 3er9B | 4qctO |  |  |
| 3bocA |  |  |  |  | 4d8gB | 1hqdA | 3fd5B |  |  |
| 3tenC |  |  |  |  | 3sl1A | 3tb3B | 4nv0B |  |  |
| 3v93F |  |  |  |  | 3ulqA | 2yn3C | 3o2eA |  |  |
| 1r23A |  |  |  |  | 2p7pF | 2qt6A | 4kwuA |  |  |
| 3u5eo |  |  |  |  | 2j46B | 4gncA | 1rkvA |  |  |
| 4a0xA |  |  |  |  | 1tc2B | 4dzrA | 4hqoA |  |  |
| 3b4nA |  |  |  |  | 3qebZ | 3powA | 4amsA |  |  |
| 2g02A |  |  |  |  | 2kfnA | 2b33B | 4qczU |  |  |
| 4a47C |  |  |  |  | 3dx5A | 2okxB | 3dgtA |  |  |
| 1a7wA |  |  |  |  | 4njjB | 2pnyA | 3fwsB |  |  |
| 4cshD |  |  |  |  | 3ounB | 3kmnB | 3hiyA |  |  |
| 2wyhA |  |  |  |  | 2pomA | 4i9xD | 2h1cA |  |  |
| 1gaxA |  |  |  |  | 1r8mE | 4hteA | 2f43B |  |  |
| 3eqtB |  |  |  |  | 4fixB | 4nt0B | 2dkgA |  |  |
| 3af5A |  |  |  |  | 4imaA | 3mhgA | 4kgmA |  |  |
| 1cprA |  |  |  |  | 3r5uB | 2yhgA | 2yp1C |  |  |
| 4h3sA |  |  |  |  | 3ke6A | 1h0hK | 3sz4A |  |  |
| 2fk6A |  |  |  |  | 2indA | 2frhA | 3en9A |  |  |
| 3p24A |  |  |  |  | 1t6bY | 4hizA | 1t3nB |  |  |
| 2yntC |  |  |  |  | 3o3hA | 4kc7B | 4b3tG |  |  |
| 4lczA |  |  |  |  | 3weiA | 3slpB | 3f1eX |  |  |
| 2y33A |  |  |  |  | 3ob8D | 3s35X | 3t5tB |  |  |
| 4l3kA |  |  |  |  | 2chrA | 3aluC | 4dzhA |  |  |
| 4levA |  |  |  |  | 1gldG | 4gejI | 2g25A |  |  |
| 4b6dA |  |  |  |  | 2qb0B | 2e26A | 3cinA |  |  |
| 3cqkA |  |  |  |  | 2zofB | 2xmoA | 3h7cX |  |  |
| 2avuF |  |  |  |  | 3mz4B | 2ozbA | 4miwA |  |  |
| 4gqtA |  |  |  |  | 4mynA | 3eifA | 1ytuB |  |  |
| 3zdrA |  |  |  |  | 1fsaB | 3fz5C | 4c3iB |  |  |
| 3b1bB |  |  |  |  | 1wljA | 1lpsA | 4knhB |  |  |
| 2j44A |  |  |  |  | 2guiA | 2j5wA | 2v54B |  |  |
| 2c2uA |  |  |  |  | 1f66G | 2xc2A | 4be1A |  |  |
| 3ea6A |  |  |  |  | 3wqoA | 2xxlB | 3s9fA |  |  |
| 1cg2D |  |  |  |  | 2w42A | 4bloL | 3gnzP |  |  |
| 3e80B |  |  |  |  | 2zxpA | 3g5cA | 2hvqA |  |  |
| 1oi0A |  |  |  |  | 2f89F | 3s4yA | 3qy7A |  |  |
| 1z3iX |  |  |  |  | 2a8pB | 2ji3A | 4a8mQ |  |  |
| 3tbgC |  |  |  |  | 3godA | 1b2lA | 4g6hA |  |  |
| 1qvnB |  |  |  |  | 3mfuA | 5enlA | 3dxjD |  |  |
| 3hftA |  |  |  |  | 4c21B | 4jx1A | 1q3uA |  |  |
| 2xumA |  |  |  |  | 3acaA | 4llsA | 4f8eB |  |  |
| 2gu2A |  |  |  |  | 3oumA | 3o4yA | 1r2rD |  |  |
| 2erpB |  |  |  |  | 2ojwC | 4n1iA | 1sxjD |  |  |
| 3ivtB |  |  |  |  | 2yl8A | 1ad5B | 3k57A |  |  |
| 3u5cb |  |  |  |  | 3a6uA | 3in9A | 4njiA |  |  |
| 4gyfA |  |  |  |  | 3zf8A | 4p0dA | 1nbmC |  |  |
| 1f4tB |  |  |  |  | 2i6qA | 1q39A | 3r9xA |  |  |
| 4cogA |  |  |  |  | 4g3hC | 1uzjC | 1xxxC |  |  |
| 7icrA |  |  |  |  | 2pokA | 4bweB | 2oekA |  |  |
| 3oruA |  |  |  |  | 3dkxA | 3zdcA | 2fp0A |  |  |
| 3rsnA |  |  |  |  | 1ii7A | 4q0zA | 3wqmA |  |  |
| 1xv2C |  |  |  |  | 3zk4B | 3griB | 2aqxA |  |  |
| 3u5em |  |  |  |  | 4dwrB | 1i9bD | 4q1vA |  |  |
| 2h39B |  |  |  |  | 3tr8B | 3bfmA | 3g1bB |  |  |
| 3w95A |  |  |  |  | 2hzyB | 3kh1A | 3kni0 |  |  |
| 2vo9B |  |  |  |  | 2yesB | 3cv1A | 1kxgA |  |  |
| 3mf2A |  |  |  |  | 2gvdA | 2zl5A | 4flxA |  |  |
| 2oodA |  |  |  |  | 4dz4B | 1wc0A | 2vf7C |  |  |
| 2xybA |  |  |  |  | 1ystM | 2yhwA | 4a01B |  |  |
| 3rmqA |  |  |  |  | 3a6kA | 4a4aA | 2yhgA |  |  |
| 2uz3D |  |  |  |  | 3q4qA | 3df6C | 4lrzD |  |  |
| 1hfeT |  |  |  |  | 1qgqA | 1ou9A | 4dn1A |  |  |
| 4gfjA |  |  |  |  | 2fc0A | 1iodA | 1musA |  |  |
| 2hzcA |  |  |  |  | 2ydtA | 3cneD | 2hsjD |  |  |
| 4fytA |  |  |  |  | 3ngfB | 4ecwA | 2p3nC |  |  |
| 2wknE |  |  |  |  | 1jstC | 3pgbA | 3r4cA |  |  |
| 2eulA |  |  |  |  | 1zm8A | 1t6mB | 2bz0A |  |  |
| 4pxyB |  |  |  |  | 1j25A | 4apmA | 1rfqB |  |  |
| 3fvzA |  |  |  |  | 3uagA | 2pvzA | 3c8vC |  |  |
| 4qvuA |  |  |  |  | 1r2mA | 4m5iA | 3s7zB |  |  |
| 3avsA |  |  |  |  | 3dt7A | 3fgeA | 4dmzB |  |  |
| 1kaeA |  |  |  |  | 4q3vC | 4jk4A | 3f1eT |  |  |
| 1f35A |  |  |  |  | 3tuxA | 3qe5B | 4kemB |  |  |
| 3a32A |  |  |  |  | 3rvrB | 1uisA | 3i6tB |  |  |
| 2ox8C |  |  |  |  | 2iryA | 4lw9D | 2jd4B |  |  |
| 2jksA |  |  |  |  | 4cswB | 1jmjA | 3cifB |  |  |
| 3hwpA |  |  |  |  | 3hqrA | 3k8kA | 2feaA |  |  |
| 2i56C |  |  |  |  | 2v8jA | 3dzmA | 2ggeD |  |  |
| 3kwoA |  |  |  |  | 4amqA | 2c1vB | 2d5fB |  |  |
| 3swnA |  |  |  |  | 4g24A | 1lwjB | 1eyzA |  |  |
| 4b29A |  |  |  |  | 2wzfA | 1bjqB | 3gieB |  |  |
| 2w88B |  |  |  |  | 1qh3B | 1i8aA | 2aekB |  |  |
| 3e9qB |  |  |  |  | 4mdaA | 2z0jC | 4m83B |  |  |
| 1jr3A |  |  |  |  | 3jyfA | 2xjpA | 3mp3C |  |  |
| 3lumA |  |  |  |  | 4phrA | 3wnkA | 3c5pD |  |  |
| 1glcF |  |  |  |  | 2o14A | 4i5nE | 2j3qA |  |  |
| 1ro5A |  |  |  |  | 3exeA | 3or3B | 1l0oB |  |  |
| 1ohtA |  |  |  |  | 3nvtA | 1qmdA | 4ncaA |  |  |
| 4aiaE |  |  |  |  | 4qrnA | 3g27A | 3u4fB |  |  |
| 3lwuA |  |  |  |  | 1xvlA | 3zniA | 2iutB |  |  |
| 1nlxK |  |  |  |  | 4a25C | 4u65E | 3bwvB |  |  |
| 3llaA |  |  |  |  | 3pztB | 2scpB | 1xdpB |  |  |
| 3lrqD |  |  |  |  | 2j3mB | 3ptyA | 3ilfA |  |  |
| 4obiA |  |  |  |  | 4lumB | 4ce5B | 4b61A |  |  |
| 1qe3A |  |  |  |  | 1bowA | 2ix0A | 4qcn4 |  |  |
| 3qndB |  |  |  |  | 3hb3A | 4hhrA | 1g8xA |  |  |
| 3ij6A |  |  |  |  | 3qxbA | 1nzyA | 3fj4A |  |  |
| 2clbM |  |  |  |  | 3jzvA | 3ttqA | 2oi2A |  |  |
| 3wojA |  |  |  |  | 1on8B | 2p6tG | 1z2oX |  |  |
| 1t2tA |  |  |  |  | 2f6kA | 2h1iB | 1golA |  |  |
| 1yo7A |  |  |  |  | 4hqnB | 3b55A | 2ix1A |  |  |
| 2y43B |  |  |  |  | 1ncyA | 4a0pA | 1zbuB |  |  |
| 3ksvA |  |  |  |  | 1khwB | 1brwA | 1su2B |  |  |
| 3h90A |  |  |  |  | 1frwA | 1ekxC | 1mc3B |  |  |
| 1vhdA |  |  |  |  | 4ilkB | 3fz0B | 3vpnA |  |  |
| 3b0xA |  |  |  |  | 3r0lD | 4b4fA | 1dekA |  |  |
| 4nefA |  |  |  |  | 2ygkA | 1b4nD | 3wbzE |  |  |
| 3u5oC |  |  |  |  | 2j0bA | 4du6C | 3ae0A |  |  |
| 2fsaA |  |  |  |  | 1rzdA | 4awyB | 4qctV |  |  |
| 1ekjG |  |  |  |  | 2dfjA | 3d8pA | 3bl5E |  |  |
| 3qbeA |  |  |  |  | 2d0cA | 2yeqB | 2oa6D |  |  |
| 2bnoB |  |  |  |  | 2fv2A | 2oznB | 1tq6A |  |  |
| 2wgqB |  |  |  |  | 3ov7A | 2wvzB | 1g9xB |  |  |
| 3m85A |  |  |  |  | 2p73A | 4nvrA | 4pl0B |  |  |
| 3w2wA |  |  |  |  | 4fm1A | 2id3A | 2npnA |  |  |
| 1nvtB |  |  |  |  | 1twfB | 2og9A | 3e3zA |  |  |
| 2np0A |  |  |  |  | 1igvA | 4ktrD | 1q3bA |  |  |
| 4f14A |  |  |  |  | 3qfmA | 1c8gA | 3eueA |  |  |
| 4ad9A |  |  |  |  | 2gvdC | 4afkA | 3djbB |  |  |
| 1r61A |  |  |  |  | 3m0vC | 3iucC | 1y9iB |  |  |
| 4lw9J |  |  |  |  | 2qb6A | 1v73A | 3hnmC |  |  |
| 2x5cB |  |  |  |  | 4qsfA | 3ouuA | 2qenA |  |  |
| 2vunB |  |  |  |  | 1v7pC | 4douA | 3mmnA |  |  |
| 4m6rB |  |  |  |  | 3s6vA | 3c7gA | 2x98A |  |  |
| 3r75B |  |  |  |  | 1k4lA | 1jn9A | 1sgjC |  |  |
| 3fm2A |  |  |  |  | 1ut5B | 1byfB | 3zthA |  |  |
| 3draB |  |  |  |  | 2whxA | 2ivzA | 3kr4C |  |  |
| 2yc5A |  |  |  |  | 1oywA | 3chjA | 4kj2N |  |  |
| 3zniM |  |  |  |  | 4ac8A | 1qi2A | 1jgtB |  |  |
| 1q7hA |  |  |  |  | 3hb3B | 1pg6A | 3m01A |  |  |
| 4c2mA |  |  |  |  | 1jaiA | 1mveA | 2zgzB |  |  |
| 3r3qA |  |  |  |  | 1nvmA | 4kakA | 2zl5A |  |  |
| 4f7oB |  |  |  |  | 1qmgA | 3gukA | 2pnqA |  |  |
| 3qlaA |  |  |  |  | 1u8xX | 4f53B | 2bx2L |  |  |
| 1q2rA |  |  |  |  | 4lt5A | 1xhbA | 3e4dB |  |  |
| 4nyuA |  |  |  |  | 1z25A | 3lorB | 2ddtA |  |  |
| 2rb4A |  |  |  |  | 2vicA | 1nqdB | 4bl0A |  |  |
| 2yi1A |  |  |  |  | 3fa3B | 4bhvD | 4bjrA |  |  |
| 4gwdB |  |  |  |  | 2nrzA | 3ut0A | 2pi4A |  |  |
| 2gyqA |  |  |  |  | 4hnoA | 4by6A | 1o4zB |  |  |
| 2f9kF |  |  |  |  | 1xzwB | 4mgqA | 4dn5A |  |  |
| 1iujB |  |  |  |  | 2ic3A | 3a68C | 1rdfE |  |  |
| 2c1dA |  |  |  |  | 4db1B | 4j6rG | 1ar1B |  |  |
| 4hdtA |  |  |  |  | 1v1fA | 3sm3A | 2z2pB |  |  |
| 3v77D |  |  |  |  | 1mavA | 1jtdB | 1dqnA |  |  |
| 1p6dA |  |  |  |  | 2g8iA | 2o39C | 4qhtC |  |  |
| 4dwcA |  |  |  |  | 3azeD | 2ddrD | 3gn6C |  |  |
| 4f9aB |  |  |  |  | 4pegC | 1uvnC | 4db8B |  |  |
| 2e6lA |  |  |  |  | 4efdF | 2jdaB | 3smlA |  |  |
| 2p18A |  |  |  |  | 4dqwB | 4c16A | 1ewkB |  |  |
| 1enrA |  |  |  |  |  | 1o8gA | 2x4dA |  |  |
| 4hexB |  |  |  |  |  | 2vs7D | 4ecwA |  |  |
| 1cu1B |  |  |  |  |  | 3c16A | 2wvlA |  |  |
| 4hc7A |  |  |  |  |  | 2eroA | 2y7fD |  |  |
| 2h6lA |  |  |  |  |  | 3pf3A | 4j6wA |  |  |
| 2f9iB |  |  |  |  |  | 2xgpB | 2p4pB |  |  |
| 3vthA |  |  |  |  |  | 2taaC | 2wtzB |  |  |
| 2pigA |  |  |  |  |  | 4kxdA | 3mx3A |  |  |
| 2ragD |  |  |  |  |  | 3fvqB | 2x03B |  |  |
| 2hsiB |  |  |  |  |  | 2p5rA | 1twyA |  |  |
| 3adrA |  |  |  |  |  | 1tjxA | 4i5qA |  |  |
| 3rpgC |  |  |  |  |  | 4av3B | 3k8kA |  |  |
| 3a1zC |  |  |  |  |  | 4k4iA | 4hvjB |  |  |
| 2a5hD |  |  |  |  |  | 1kfqB | 4la6A |  |  |
| 2pptA |  |  |  |  |  | 1smvC | 4hchA |  |  |
| 1x6mB |  |  |  |  |  | 3q7hG | 1zszC |  |  |
| 1i1iP |  |  |  |  |  | 3b4nA | 3f1fI |  |  |
| 3htrA |  |  |  |  |  | 3edyA | 2ofxB |  |  |
| 3g45A |  |  |  |  |  | 2cdpA | 1xqaA |  |  |
| 4o2iA |  |  |  |  |  | 3na6A | 3oi7D |  |  |
| 4mthA |  |  |  |  |  | 4bb9A | 1ta0A |  |  |
| 3u5gd |  |  |  |  |  | 4evhA | 4b3mI |  |  |
| 1au1B |  |  |  |  |  | 2xt6A | 4m30A |  |  |
| 1cnqA |  |  |  |  |  | 1y1aB | 3kkoA |  |  |
| 2ch9A |  |  |  |  |  | 2nxqA | 2vhxF |  |  |
| 4msjA |  |  |  |  |  | 4l9oA | 3wrxC |  |  |
| 3eyyB |  |  |  |  |  | 3dpgB | 4iedC |  |  |
| 2chuA |  |  |  |  |  | 2xhjA | 3jvtB |  |  |
| 3o47A |  |  |  |  |  | 4ihbE | 3e2dA |  |  |
| 1rp0B |  |  |  |  |  | 2c8sA | 4b3mB |  |  |
| 4knkB |  |  |  |  |  | 3fd2A | 3b8iF |  |  |
| 3jxpA |  |  |  |  |  | 4ej7A | 1h7qA |  |  |
| 3t01A |  |  |  |  |  | 2essA | 3knkZ |  |  |
| 4hyfB |  |  |  |  |  | 2dcjA | 2a5zC |  |  |
| 1iwlA |  |  |  |  |  | 4ln1B | 4kj9D |  |  |
| 4lfyB |  |  |  |  |  | 3t1gA | 2qm1B |  |  |
| 4gx0B |  |  |  |  |  | 3hzbD | 1vtnC |  |  |
| 2i2xM |  |  |  |  |  | 1x8kA | 8iciA |  |  |
| 2einB |  |  |  |  |  | 3attA | 2qv7A |  |  |
| 3gn5A |  |  |  |  |  | 4olkB | 2x0qA |  |  |
| 2i0oA |  |  |  |  |  | 4lqrA | 3ip4B |  |  |
| 3qnvA |  |  |  |  |  | 2w4yC | 4m5nB |  |  |
| 1y7pB |  |  |  |  |  | 2pplA | 1u3cA |  |  |
| 1mr1C |  |  |  |  |  | 3k9jA | 3rmwA |  |  |
| 1ybqA |  |  |  |  |  | 4jqpA | 1u7pA |  |  |
| 4k2hD |  |  |  |  |  | 3ufkA | 4nstD |  |  |
| 2fvmB |  |  |  |  |  | 1cjyA | 4awyB |  |  |
| 1r43A |  |  |  |  |  | 1gxoA | 3s14A |  |  |
| 3qbuD |  |  |  |  |  | 4m0kC | 3w40B |  |  |
| 1ck7A |  |  |  |  |  | 2zexA | 3tepA |  |  |
| 2dgeA |  |  |  |  |  | 3axhA | 2wvaX |  |  |
| 3coqB |  |  |  |  |  | 1uhaA | 4qdgB |  |  |
| 1io0A |  |  |  |  |  | 2rauA | 3rfeA |  |  |
| 3vqjA |  |  |  |  |  | 3v5uA | 3t1rC |  |  |
| 3dzaC |  |  |  |  |  | 4n2pA | 2jkgA |  |  |
| 4kjmA |  |  |  |  |  | 1guiA | 4ggmX |  |  |
| 4lmgB |  |  |  |  |  | 1de4I | 4gu5B |  |  |
| 4pysA |  |  |  |  |  | 2qp2A | 2zscA |  |  |
| 2vkrD |  |  |  |  |  | 3dt5A | 4kj6U |  |  |
| 1b20A |  |  |  |  |  | 2qtwB | 1k77A |  |  |
| 1pegA |  |  |  |  |  | 1ga2B | 1k68A |  |  |
| 4msxA |  |  |  |  |  | 1v7wA | 2isiA |  |  |
| 4enlA |  |  |  |  |  | 3rqiA | 4bbjA |  |  |
| 2ek0A |  |  |  |  |  | 4infA | 4q86H |  |  |
| 2vh3B |  |  |  |  |  | 4f9jA | 3u7qD |  |  |
| 2yz5B |  |  |  |  |  | 3pp5A | 1nziB |  |  |
| 1ef0A |  |  |  |  |  | 4cc1A | 4hubN |  |  |
| 3rxzC |  |  |  |  |  | 2y3cA | 3ii9C |  |  |
| 4q8gB |  |  |  |  |  | 2yv9A | 1wywB |  |  |
| 1ak0A |  |  |  |  |  | 3vj9A | 3shxA |  |  |
| 4fc5C |  |  |  |  |  | 3irhC | 4kiyV |  |  |
| 4guaC |  |  |  |  |  | 2vveA | 4gbfA |  |  |
| 4filD |  |  |  |  |  | 3hjbB | 1u02A |  |  |
| 3f6hB |  |  |  |  |  | 1yvuA | 1rkqA |  |  |
| 4lh8A |  |  |  |  |  | 4b5wA | 3o98B |  |  |
| 4nvv6 |  |  |  |  |  | 3en8A | 1bl3C |  |  |
| 2exuA |  |  |  |  |  | 3goeA | 2d0bA |  |  |
| 3floD |  |  |  |  |  | 1axkB | 2j7nA |  |  |
| 1mkmB |  |  |  |  |  | 4hsqA | 1iruY |  |  |
| 4kjgA |  |  |  |  |  | 1x7iB | 4jejA |  |  |
| 1hkkA |  |  |  |  |  | 1up8C | 3u0oB |  |  |
| 4i14A |  |  |  |  |  | 3uowA | 2i71B |  |  |
| 3llxA |  |  |  |  |  | 4mivA | 3ozgA |  |  |
| 2bo0A |  |  |  |  |  | 4ii3A | 1nmpE |  |  |
| 3ht2C |  |  |  |  |  | 4hpnA | 3bznA |  |  |
| 2fbhA |  |  |  |  |  | 4g62B | 2p5eB |  |  |
| 4i2xB |  |  |  |  |  | 3pvhA | 3ef1A |  |  |
| 1pszA |  |  |  |  |  | 1wvmB | 3ktnA |  |  |
| 4bmjC |  |  |  |  |  | 3gazA | 2ia5H |  |  |
| 2hf9A |  |  |  |  |  | 2ea7A | 2d32A |  |  |
| 4ojzA |  |  |  |  |  | 4p99C | 1y37B |  |  |
| 3ga3A |  |  |  |  |  | 4kxvA | 3r7wC |  |  |
| 3sjaB |  |  |  |  |  | 2plyA | 3na5A |  |  |
| 2vrzA |  |  |  |  |  | 3pe0A | 2j5vA |  |  |
| 3fjuB |  |  |  |  |  | 3w7aC | 3kd3B |  |  |
| 1onsA |  |  |  |  |  | 2q1fA | 3q6jA |  |  |
| 4k4tA |  |  |  |  |  | 3c5iD | 1j7lA |  |  |
| 3irbA |  |  |  |  |  | 1ji1A | 1yunB |  |  |
| 4ql5B |  |  |  |  |  | 3dssB | 3mmhB |  |  |
| 4l6hA |  |  |  |  |  | 3aujA | 4aq4A |  |  |
| 2d0wB |  |  |  |  |  | 2p1rA | 4en4A |  |  |
| 2j0eB |  |  |  |  |  | 3uvfA | 1obgA |  |  |
| 1rqgA |  |  |  |  |  | 1espA | 2br3F |  |  |
| 1b71A |  |  |  |  |  | 6dfrA | 2v4oD |  |  |
| 1qp9A |  |  |  |  |  | 3m0jA | 4omfA |  |  |
| 3e6uA |  |  |  |  |  | 2pjpA | 3k5hA |  |  |
| 2ybpB |  |  |  |  |  | 4n1gA | 3ivsA |  |  |
| 1zkpD |  |  |  |  |  | 3uf5B | 1s4eG |  |  |
| 3e7gC |  |  |  |  |  | 3kstB | 1nrjB |  |  |
| 1u2wB |  |  |  |  |  | 4lxzC | 2a41B |  |  |
| 3cp0A |  |  |  |  |  | 2rjiA | 4nmuD |  |  |
| 1xx4A |  |  |  |  |  | 2es2A | 2b8jB |  |  |
| 4dr8A |  |  |  |  |  | 1pk8A | 2xkbI |  |  |
| 1ta9B |  |  |  |  |  | 4oyxA | 2huhA |  |  |
| 1gpcA |  |  |  |  |  | 1wwsC | 2pa4D |  |  |
| 4a6eA |  |  |  |  |  | 4fl4I | 2dsyB |  |  |
| 3luoA |  |  |  |  |  | 3fybA | 4cc7M |  |  |
| 3h0nA |  |  |  |  |  | 1p3hB | 4fh5A |  |  |
| 3q87A |  |  |  |  |  | 2fycB | 2xt6A |  |  |
| 3g8rA |  |  |  |  |  | 3hm2G | 4jasA |  |  |
| 3i9fB |  |  |  |  |  | 3zr5A | 1w23B |  |  |
| 3zxoB |  |  |  |  |  | 3fvzA | 4idnB |  |  |
| 2g7zB |  |  |  |  |  | 1s99A | 3swiA |  |  |
| 2x3yA |  |  |  |  |  | 3toyC | 1g69B |  |  |
| 3mx2B |  |  |  |  |  | 1y6oB | 2xspA |  |  |
| 1z1nX |  |  |  |  |  | 1ee6A | 2f1fA |  |  |
| 4mi5A |  |  |  |  |  | 3pddA | 1gqcA |  |  |
| 1dcqA |  |  |  |  |  | 4cfrB | 1wzcB |  |  |
| 1o7dA |  |  |  |  |  | 2i52F | 2xdgA |  |  |
| 4l44A |  |  |  |  |  | 4ibrA | 2o3bB |  |  |
| 2zzeA |  |  |  |  |  | 1v3wA | 4oz6A |  |  |
| 3iibA |  |  |  |  |  | 4aylA | 1w85C |  |  |
| 2hpiA |  |  |  |  |  | 3bbyA | 4dscB |  |  |
| 1dceB |  |  |  |  |  | 4axnB | 4l8nA |  |  |
| 2pdoD |  |  |  |  |  | 4dktA | 3attA |  |  |
| 1zh1A |  |  |  |  |  | 1m9iA | 1v26B |  |  |
| 1jr3E |  |  |  |  |  | 3rmkE | 4j03A |  |  |
| 3wrgA |  |  |  |  |  | 4l0pB | 2q3wA |  |  |
| 2dfyX |  |  |  |  |  | 3lumA | 3kx2A |  |  |
| 3cngC |  |  |  |  |  | 3r4iA | 3ufkA |  |  |
| 3eqnB |  |  |  |  |  | 2odiB | 4e7pB |  |  |
| 4e45N |  |  |  |  |  | 3fz3F | 3wxmA |  |  |
| 4lu3A |  |  |  |  |  | 1o7lD | 2gslF |  |  |
| 1j6wA |  |  |  |  |  | 1j5uA | 3reuA |  |  |
| 4c3iJ |  |  |  |  |  | 2e9bB | 2qndA |  |  |
| 3hmfA |  |  |  |  |  | 4e5uB | 3e4oB |  |  |
| 3t6pA |  |  |  |  |  | 2wz8A | 3pmgB |  |  |
| 2iv0A |  |  |  |  |  | 3wu2O | 3vytC |  |  |
| 2hekB |  |  |  |  |  | 1b94A | 2jf5A |  |  |
| 4iloA |  |  |  |  |  | 2pc6C | 2r5wB |  |  |
| 1k83I |  |  |  |  |  | 3o4pA | 13pkA |  |  |
| 4nn2A |  |  |  |  |  | 4jzbB | 1jlsB |  |  |
| 1snnB |  |  |  |  |  | 4mrcA | 1ga2B |  |  |
| 1k2yX |  |  |  |  |  | 4l75A | 4qjbA |  |  |
| 3q03B |  |  |  |  |  | 1h6gA | 1r0zC |  |  |
| 3phxA |  |  |  |  |  | 3b0xA | 2yzjC |  |  |
| 4a2cB |  |  |  |  |  | 1mj2B | 1mumA |  |  |
| 1hr6F |  |  |  |  |  | 2fxfA | 3hwx1 |  |  |
| 4c4aA |  |  |  |  |  | 4ci7A | 2alfA |  |  |
| 3ts2B |  |  |  |  |  | 1bgpA | 1gkbB |  |  |
| 2zj7A |  |  |  |  |  | 4lvnA | 3n84C |  |  |
| 3sd9A |  |  |  |  |  | 3r6qH | 1w0nA |  |  |
| 1oqjA |  |  |  |  |  | 4lq0A | 2o35B |  |  |
| 2xvaB |  |  |  |  |  | 3wq7A | 3tvaA |  |  |
| 2dkcA |  |  |  |  |  | 4k70A | 4j13B |  |  |
| 2d5mA |  |  |  |  |  | 3ipkA | 4hppA |  |  |
| 1vq0A |  |  |  |  |  | 4qu6A | 2hawB |  |  |
| 2az4B |  |  |  |  |  | 3mkzA | 2pp3C |  |  |
| 2ev6B |  |  |  |  |  | 4i2nD | 4m4qA |  |  |
| 3uedC |  |  |  |  |  | 2wm4A | 1htwA |  |  |
| 1iahB |  |  |  |  |  | 2hcnA | 3q7eA |  |  |
| 3e1zA |  |  |  |  |  | 2wgqB | 3o3fA |  |  |
| 1n08B |  |  |  |  |  | 1kitA | 4jn9A |  |  |
| 3n3uA |  |  |  |  |  | 4n0oC | 3mwbB |  |  |
| 4u5gA |  |  |  |  |  | 2xfvA | 1hbnF |  |  |
| 3g8qA |  |  |  |  |  | 3t41A | 2v9pA |  |  |
| 3rf5A |  |  |  |  |  | 2cmzC | 1xd9A |  |  |
| 3favC |  |  |  |  |  | 2np0A | 4l80C |  |  |
| 1lbuA |  |  |  |  |  | 1qh4D | 4k99A |  |  |
| 2yb5F |  |  |  |  |  | 4jf7A | 4gedA |  |  |
| 3isoA |  |  |  |  |  | 4ggqA | 3dlzA |  |  |
| 3m9gA |  |  |  |  |  | 3r3tA | 1xbtA |  |  |
| 3qfkA |  |  |  |  |  | 4m8dB | 1qb7A |  |  |
| 2vjfA |  |  |  |  |  | 3sucA | 2od0A |  |  |
| 3gucA |  |  |  |  |  | 3ck9B | 4ii3A |  |  |
| 3o0fA |  |  |  |  |  | 4km6A | 2qlxB |  |  |
| 3h5aC |  |  |  |  |  | 2ex5B | 1wvmB |  |  |
| 2vcgD |  |  |  |  |  | 1hmwA | 2o8dB |  |  |
| 2yheA |  |  |  |  |  | 1c8nC | 3ozxB |  |  |
| 2xb1C |  |  |  |  |  | 1nbwA | 1tqyC |  |  |
| 3ljuX |  |  |  |  |  | 3bjuA | 3fp2A |  |  |
| 1ty2A |  |  |  |  |  | 1nubA | 3fe4B |  |  |
| 3lqiC |  |  |  |  |  | 3mq7E | 2q0eA |  |  |
| 4axdA |  |  |  |  |  | 4p57B | 1knyB |  |  |
| 1sw4A |  |  |  |  |  | 3etoB | 2plyA |  |  |
| 3ct8A |  |  |  |  |  | 1t5dX | 1f5nA |  |  |
| 1odhA |  |  |  |  |  | 4ghcB | 4lsbB |  |  |
| 3c9fB |  |  |  |  |  | 4dnnB | 3q46A |  |  |
| 4c3bF |  |  |  |  |  | 3wfbC | 4nclA |  |  |
| 1de5A |  |  |  |  |  | 3aqjA | 3rv0B |  |  |
| 3bvoA |  |  |  |  |  | 3vtoB | 1q9kB |  |  |
| 4iuvA |  |  |  |  |  | 2o4vB | 2i4oA |  |  |
| 4c5yA |  |  |  |  |  | 2bh1A | 2pjpA |  |  |
| 3czxC |  |  |  |  |  | 4plrB | 4ajrA |  |  |
| 4bwzA |  |  |  |  |  | 3u39C | 2vrnA |  |  |
| 1btgB |  |  |  |  |  | 3gflA | 4un4B |  |  |
| 2dphB |  |  |  |  |  | 3uxfA | 2cjeA |  |  |
| 2pgqB |  |  |  |  |  | 2i6hB | 1pfkB |  |  |
| 3m7kA |  |  |  |  |  | 4iyqA | 4e2pA |  |  |
| 1ldjB |  |  |  |  |  | 3jq1B | 3fnbA |  |  |
| 3n9mA |  |  |  |  |  | 3nqnA | 2hfzA |  |  |
| 4l9pB |  |  |  |  |  | 4m7kH | 2uxqA |  |  |
| 2dvtA |  |  |  |  |  | 2gpjA | 3pt1A |  |  |
| 2v9kA |  |  |  |  |  | 2owlA | 2pzaA |  |  |
| 1irxB |  |  |  |  |  | 1t9hA | 3ssoD |  |  |
| 2p50A |  |  |  |  |  | 4l03C | 3l8fA |  |  |
| 3nybB |  |  |  |  |  | 2wjsA | 1vrpA |  |  |
| 3luuA |  |  |  |  |  | 3auaB | 2ajrB |  |  |
| 2qpxA |  |  |  |  |  | 1snnA | 3r2uD |  |  |
| 3icjA |  |  |  |  |  | 1rc8A | 3eg7B |  |  |
| 3g4hA |  |  |  |  |  | 3v6iA | 3hm2G |  |  |
| 4m3oA |  |  |  |  |  | 3dr2A | 1yfrB |  |  |
| 3a1bA |  |  |  |  |  | 4nasB | 1wuuA |  |  |
| 4af1A |  |  |  |  |  | 4i9fB | 3klcB |  |  |
| 3vpbE |  |  |  |  |  | 4ag4A | 4oeiA |  |  |
| 3mhsA |  |  |  |  |  | 2pyhB | 4f5dB |  |  |
| 1oj7D |  |  |  |  |  | 3d7kA | 3gcmB |  |  |
| 2j6aA |  |  |  |  |  | 4dk7A | 1yw0D |  |  |
| 1u0aD |  |  |  |  |  | 4fa4B | 2gmsA |  |  |
| 2p2lC |  |  |  |  |  | 2vmaA | 4m33A |  |  |
| 3q3qA |  |  |  |  |  | 3w5mA | 3gsiA |  |  |
| 3p1vA |  |  |  |  |  | 3hiiA | 2awoA |  |  |
| 3ky9B |  |  |  |  |  | 1xzoA | 3aq0C |  |  |
| 4lqxB |  |  |  |  |  | 3zo9B | 2ppqA |  |  |
| 3orjA |  |  |  |  |  | 3tb4A | 1yvhA |  |  |
| 3qgvA |  |  |  |  |  | 2yknA | 1esnA |  |  |
| 3ramA |  |  |  |  |  | 3fg3A | 3uoaC |  |  |
| 2igiA |  |  |  |  |  | 4c07A | 3kzqB |  |  |
| 4cc9B |  |  |  |  |  | 2qm3A | 4azwA |  |  |
| 3lcnB |  |  |  |  |  | 2io4B | 3uzrA |  |  |
| 3ujzA |  |  |  |  |  | 2btwA | 1ze1B |  |  |
| 4czwA |  |  |  |  |  | 2d73A | 2bmuB |  |  |
| 4fajA |  |  |  |  |  | 2oa8B | 2pywA |  |  |
| 2v1zA |  |  |  |  |  | 4lmhD | 1q4rA |  |  |
| 2gx8C |  |  |  |  |  | 4hjfA | 1dgmA |  |  |
| 2obaC |  |  |  |  |  | 3ci0K | 3n3tB |  |  |
| 1q7lA |  |  |  |  |  | 2jfpB | 3ffuA |  |  |
| 3pe8A |  |  |  |  |  | 1qcnB | 3wcwB |  |  |
| 3r3rA |  |  |  |  |  | 1ovaA | 4mhxB |  |  |
| 3mbgC |  |  |  |  |  | 4mbzA | 1qpbB |  |  |
| 3di4A |  |  |  |  |  | 3gv5B | 3vc2D |  |  |
| 2gpyB |  |  |  |  |  | 2zbaA | 1v8vA |  |  |
| 3ce9C |  |  |  |  |  | 1w2mF | 1i7qA |  |  |
| 3efoB |  |  |  |  |  | 4immA | 3aqbD |  |  |
| 4bz5B |  |  |  |  |  | 1szoJ | 2gfqA |  |  |
| 3t3wF |  |  |  |  |  | 4dh2B | 4fk1D |  |  |
| 2owbA |  |  |  |  |  | 3u7zA | 4bwrA |  |  |
| 4g3mD |  |  |  |  |  | 4ex8A | 2qvhA |  |  |
| 1qipD |  |  |  |  |  | 3ojcA | 3onnA |  |  |
| 1i6nA |  |  |  |  |  | 3r8yD | 3zkbD |  |  |
| 2a5vA |  |  |  |  |  | 4ggfL | 3qr3B |  |  |
| 3rcqA |  |  |  |  |  | 4aq0A | 1i3mA |  |  |
| 1t0bG |  |  |  |  |  | 3vu1B | 1wbbA |  |  |
| 2h1nA |  |  |  |  |  | 4aslA | 4ijxA |  |  |
| 3goxA |  |  |  |  |  | 4f3rB | 3qf7A |  |  |
| 2er8D |  |  |  |  |  | 3a8rB | 1ouoA |  |  |
| 1qx0A |  |  |  |  |  | 4bxoB | 3hfwA |  |  |
| 2nxfA |  |  |  |  |  | 2zzjA | 2okvB |  |  |
| 3sjpA |  |  |  |  |  | 4khoA | 3v6jJ |  |  |
| 2b9dA |  |  |  |  |  | 4it5D | 4cvoA |  |  |
| 1a6fA |  |  |  |  |  | 4a42A | 4i27A |  |  |
| 1shwA |  |  |  |  |  | 4fouA | 3bpdF |  |  |
| 2nlyA |  |  |  |  |  | 2afbA | 3gveA |  |  |
| 1cjvC |  |  |  |  |  | 2r01A | 4brsA |  |  |
| 3bpuA |  |  |  |  |  | 4a6oA | 3hb0C |  |  |
| 3h1mA |  |  |  |  |  | 4ic5B | 4fmaB |  |  |
| 1zzmA |  |  |  |  |  | 4lfyB | 4i3mA |  |  |
| 4a3nA |  |  |  |  |  | 4gx0B | 3ob8C |  |  |
| 1lfwA |  |  |  |  |  | 3by6C | 4hjhA |  |  |
| 1ekmA |  |  |  |  |  | 2c60A | 4ak6A |  |  |
| 2e18A |  |  |  |  |  | 3mzoB | 1lnzA |  |  |
| 4gndC |  |  |  |  |  | 4jsoA | 4g7hP |  |  |
| 2j6xD |  |  |  |  |  | 3qwuA | 2ik0B |  |  |
| 3fl2A |  |  |  |  |  | 3fcxB | 1y8aA |  |  |
| 4lqfA |  |  |  |  |  | 1te2B | 1rifB |  |  |
| 2wkxA |  |  |  |  |  | 2wl3A | 3w2wA |  |  |
| 4o64C |  |  |  |  |  | 3jqxC | 3oghB |  |  |
| 3t9kA |  |  |  |  |  | 4bxoA | 3hrdA |  |  |
| 3cx3A |  |  |  |  |  | 1q23A | 3tawA |  |  |
| 4cthA |  |  |  |  |  | 3l2nA | 1s5jA |  |  |
| 4k1tC |  |  |  |  |  | 2bueA | 3vkkA |  |  |
| 2z2yA |  |  |  |  |  | 4mspA | 3q1oA |  |  |
| 3pm6A |  |  |  |  |  | 4oa3A | 1nr9D |  |  |
| 3h6tB |  |  |  |  |  | 1ff5B | 3pegA |  |  |
| 3hugJ |  |  |  |  |  | 1ck7A | 4l6aA |  |  |
| 4lgrA |  |  |  |  |  | 4dlkA | 3r10A |  |  |
| 2vqxA |  |  |  |  |  | 2vl8A | 3sucA |  |  |
| 3mp2A |  |  |  |  |  | 1i9zA | 3gf0A |  |  |
| 4muqA |  |  |  |  |  | 4ac8A | 2dh4A |  |  |
| 1xvxA |  |  |  |  |  | 1nnlB | 3zxsA |  |  |
| 1qwyA |  |  |  |  |  | 2x49A | 1t57B |  |  |
| 3c37A |  |  |  |  |  | 2oblA | 4e8lC |  |  |
| 2vqgH |  |  |  |  |  | 2ygmB | 1rvcB |  |  |
| 3caoA |  |  |  |  |  | 1os1A | 3qy8A |  |  |
| 4ka8A |  |  |  |  |  | 1t61C | 3pu9A |  |  |
| 3ohiA |  |  |  |  |  | 1lkvX | 3o8rA |  |  |
| 2ygtA |  |  |  |  |  | 3hbzA | 3rplB |  |  |
| 2q1zB |  |  |  |  |  | 1ug9A | 3uplA |  |  |
| 2c6nA |  |  |  |  |  | 1fyzA | 1p18B |  |  |
| 4kepA |  |  |  |  |  | 4mkmA | 3vzxB |  |  |
| 3e7lC |  |  |  |  |  | 2wg7A | 3knk5 |  |  |
| 3dfkA |  |  |  |  |  | 3hlyB | 1rzrA |  |  |
| 2o6iA |  |  |  |  |  | 1bagA | 2fglB |  |  |
| 2rccC |  |  |  |  |  | 4lplA | 4eoyC |  |  |
| 3odcB |  |  |  |  |  | 2v9mA | 3c6aA |  |  |
| 1fioA |  |  |  |  |  | 3odtB | 4lemC |  |  |
| 1j2xA |  |  |  |  |  | 4p5fA | 1yxoB |  |  |
| 3m4gF |  |  |  |  |  | 3wl4A | 3nzgD |  |  |
| 4hp3C |  |  |  |  |  | 3rrwA | 1wvcA |  |  |
| 1ci3M |  |  |  |  |  | 2xzvA | 3n4fA |  |  |
| 4qn1A |  |  |  |  |  | 3rvvA | 1gqyB |  |  |
| 3lu2B |  |  |  |  |  | 2e1qD | 4fppB |  |  |
| 3poaA |  |  |  |  |  | 2iufE | 3rt0A |  |  |
| 3kdeC |  |  |  |  |  | 2wkpA | 4dgtB |  |  |
| 2wjyA |  |  |  |  |  | 4jo5A | 4hv6B |  |  |
| 3zbwA |  |  |  |  |  | 4gkbC | 4c12A |  |  |
| 4fo9A |  |  |  |  |  | 2r16A | 3cawB |  |  |
| 3qahA |  |  |  |  |  | 1k3iA | 1nuiA |  |  |
| 3gyyD |  |  |  |  |  | 2wimA | 3ohaA |  |  |
| 2z72A |  |  |  |  |  | 4jclA | 4fk9A |  |  |
| 2fidB |  |  |  |  |  | 2durA | 1xrjB |  |  |
| 3aiiA |  |  |  |  |  | 4mhvA | 2go7C |  |  |
| 2aa4A |  |  |  |  |  | 3tr5A | 1vx8H |  |  |
| 1mh2A |  |  |  |  |  | 1nolA | 2xtoB |  |  |
| 4lx9A |  |  |  |  |  | 4aw7A | 3tp4B |  |  |
| 2vhfA |  |  |  |  |  | 4cidA | 1n67A |  |  |
| 3iabB |  |  |  |  |  | 3qleA | 4e6eA |  |  |
| 3qsvA |  |  |  |  |  | 2r2iA | 4i8eX |  |  |
| 3hnaA |  |  |  |  |  | 3rq0A | 3vh7E |  |  |
| 2i9wA |  |  |  |  |  | 3n9sA | 1h7uA |  |  |
| 3m6iB |  |  |  |  |  | 3o3gA | 2izsA |  |  |
| 1zdeA |  |  |  |  |  | 3hl0A | 3dd2H |  |  |
| 2xoyB |  |  |  |  |  | 1w5dA | 1iruC |  |  |
| 1r4vA |  |  |  |  |  | 1z29A | 2qgyA |  |  |
| 2cs7A |  |  |  |  |  | 1bobA | 2x6vA |  |  |
| 2xcmF |  |  |  |  |  | 3pz0B | 2iqqB |  |  |
| 1wbqA |  |  |  |  |  | 3agnA | 3zo9B |  |  |
| 2gviA |  |  |  |  |  | 3smnA | 3n1gA |  |  |
| 1dk4B |  |  |  |  |  | 1j83B | 3zx4B |  |  |
| 3kyaA |  |  |  |  |  | 3hrdE | 2fueA |  |  |
| 1gleG |  |  |  |  |  | 2vr0B | 3bqbX |  |  |
| 2xqnT |  |  |  |  |  | 3iayA | 3q15D |  |  |
| 3u3dA |  |  |  |  |  | 4b0aA | 2xcwA |  |  |
| 4cj1A |  |  |  |  |  | 3ia7A | 3f2bA |  |  |
| 4a7zA |  |  |  |  |  | 1yfqA | 4qcvY |  |  |
| 1hp7A |  |  |  |  |  | 1oupA | 4l3aA |  |  |
| 3k6jA |  |  |  |  |  | 3c25A | 3bm4B |  |  |
| 2q7sB |  |  |  |  |  | 3qfeA | 3auoA |  |  |
| 2z94A |  |  |  |  |  | 3cvgB | 2v5kA |  |  |
| 3d00A |  |  |  |  |  | 1hdfA | 3vczB |  |  |
| 1noyB |  |  |  |  |  | 3sqgA | 2fq1A |  |  |
| 1lhnA |  |  |  |  |  | 3zypA | 3jysA |  |  |
| 1zfnA |  |  |  |  |  | 2zzvA | 4jbtA |  |  |
| 1zkjA |  |  |  |  |  | 2cyyA | 4immA |  |  |
| 3n55A |  |  |  |  |  | 3ecqB | 1fhvA |  |  |
| 3t3oA |  |  |  |  |  | 3bdvA | 1q3qC |  |  |
| 3m52A |  |  |  |  |  | 4jduA | 2iyyA |  |  |
| 2owoA |  |  |  |  |  | 4mboA | 3orqA |  |  |
| 3li2A |  |  |  |  |  | 2i44A | 4ktzA |  |  |
| 2ar3B |  |  |  |  |  | 3n91A | 3r3sB |  |  |
| 2z58B |  |  |  |  |  | 3qr0A | 3njlA |  |  |
| 2zsgA |  |  |  |  |  | 1fw2A | 1x07A |  |  |
| 2x80A |  |  |  |  |  | 4g2sB | 3slrA |  |  |
| 4olsA |  |  |  |  |  | 2vy0A | 3ppiB |  |  |
| 4me3A |  |  |  |  |  | 3rl5A | 3wewA |  |  |
| 1udvA |  |  |  |  |  | 1nkqA | 4j5iF |  |  |
| 1hxrB |  |  |  |  |  | 3exmA | 3c9uB |  |  |
| 2xy4A |  |  |  |  |  | 3oqqA | 2gqsA |  |  |
| 1taqA |  |  |  |  |  | 3nurA | 3i8bA |  |  |
| 2xsnD |  |  |  |  |  | 4efzA | 3gxzB |  |  |
| 4l63A |  |  |  |  |  | 3dedC | 3ulkA |  |  |
| 3khiA |  |  |  |  |  | 4luqB | 1yl5A |  |  |
| 1l0yA |  |  |  |  |  | 4ecgA | 3ksqB |  |  |
| 1hk8A |  |  |  |  |  | 4kh9A | 3ombA |  |  |
| 2imsA |  |  |  |  |  | 1rk8A | 4q4aA |  |  |
| 1waaF |  |  |  |  |  | 2d4cA | 3qvqA |  |  |
| 1p9eB |  |  |  |  |  | 3qc6X | 2rioA |  |  |
| 3ayvD |  |  |  |  |  | 2xhiA | 3t6rA |  |  |
| 2ejcA |  |  |  |  |  | 1fxwA | 3dylB |  |  |
| 1c8yA |  |  |  |  |  | 2vn6B | 4o2wA |  |  |
| 4hx3C |  |  |  |  |  | 3h4sE | 3fdgB |  |  |
| 1x1vB |  |  |  |  |  | 2yocB | 4lpsA |  |  |
| 1q1yA |  |  |  |  |  | 2wbfX | 3qqyA |  |  |
| 1c3rA |  |  |  |  |  | 1yiqA | 1w49B |  |  |
| 1zzhB |  |  |  |  |  | 4k25A | 3bk5A |  |  |
| 3qdhA |  |  |  |  |  | 4m7nA | 3aqlA |  |  |
| 4p3xA |  |  |  |  |  | 3hfjB | 3ez6B |  |  |
| 2ushB |  |  |  |  |  | 1lvuD | 3exfC |  |  |
| 1eh6A |  |  |  |  |  | 3s6jE | 3e5zA |  |  |
| 2yv5A |  |  |  |  |  | 2jalB | 4f0qB |  |  |
| 1kq0A |  |  |  |  |  | 2zuyA | 4m69A |  |  |
| 3m7pA |  |  |  |  |  | 4ki8F | 3da3A |  |  |
| 4jqpB |  |  |  |  |  | 2qt7B | 1iwlA |  |  |
| 4fglD |  |  |  |  |  | 4nehA | 2oy3A |  |  |
| 3sb5C |  |  |  |  |  | 1ve5A | 3w6nB |  |  |
| 4mtuA |  |  |  |  |  | 3nngA | 1wnoB |  |  |
| 1gupA |  |  |  |  |  | 3ktbD | 1nulA |  |  |
| 3a9lB |  |  |  |  |  | 4g01A | 2flcA |  |  |
| 2y20F |  |  |  |  |  | 2g0yA | 4b3mQ |  |  |
| 1v0dA |  |  |  |  |  | 1t0iA | 3ephA |  |  |
| 2x7mA |  |  |  |  |  | 1w07A | 2plsI |  |  |
| 1vq2A |  |  |  |  |  | 2cn3B | 2aruA |  |  |
| 3urzB |  |  |  |  |  | 2xvtC | 3v94G |  |  |
| 3plwA |  |  |  |  |  | 2bq4B | 3uulA |  |  |
| 3ifuA |  |  |  |  |  | 4mr0A | 4osqA |  |  |
| 2c1gA |  |  |  |  |  | 3dxpA | 4hubZ |  |  |
| 3qdfA |  |  |  |  |  | 2qimA | 2x1aA |  |  |
| 3l11A |  |  |  |  |  | 3e79A | 4ag5A |  |  |
| 4fukA |  |  |  |  |  | 2xqoA | 2om6B |  |  |
| 3tdpA |  |  |  |  |  | 1wmeA | 3q22A |  |  |
| 4k7dB |  |  |  |  |  | 3b2zF | 3m6zA |  |  |
| 4kfvA |  |  |  |  |  | 1eggB | 2p97A |  |  |
| 1vj0A |  |  |  |  |  | 4nhdD | 3vpbB |  |  |
| 3c2sA |  |  |  |  |  | 2w3oA | 4kegA |  |  |
| 1k9zA |  |  |  |  |  | 4mz0A | 1w1wC |  |  |
| 4jjjA |  |  |  |  |  | 4petA | 3kkiA |  |  |
| 1zfpE |  |  |  |  |  | 3jtxB | 3gahA |  |  |
| 1ddzB |  |  |  |  |  | 1ohzB | 1o6tA |  |  |
| 2q0yA |  |  |  |  |  | 1uhnA | 3hs0F |  |  |
| 3mduA |  |  |  |  |  | 3ak5B | 2yk0A |  |  |
| 3mruB |  |  |  |  |  | 2xqxA | 2w8dB |  |  |
| 2y3dA |  |  |  |  |  | 1zud3 | 1ohfC |  |  |
| 2h6tA |  |  |  |  |  | 3s4lA | 2vu9A |  |  |
| 1wdkA |  |  |  |  |  | 3lybB | 3wryB |  |  |
| 3ly0B |  |  |  |  |  | 2eufA | 3b1vA |  |  |
| 2eg3A |  |  |  |  |  | 4bgbA | 3fvdB |  |  |
| 3ztvA |  |  |  |  |  | 2xbfA | 3gocA |  |  |
| 2xs7A |  |  |  |  |  | 2q1cX | 1os1A |  |  |
| 2nx8A |  |  |  |  |  | 2pr7B | 3iceC |  |  |
| 4c7aA |  |  |  |  |  | 2w1uB | 4kavA |  |  |
| 3rysA |  |  |  |  |  | 1ru4A | 3ie7A |  |  |
| 1wczA |  |  |  |  |  | 3dasA | 2r25B |  |  |
| 4jixA |  |  |  |  |  | 1dmuA | 3s5fB |  |  |
| 1ud9A |  |  |  |  |  | 2az1D | 3ic4A |  |  |
| 2fqpC |  |  |  |  |  | 1qlsA | 3bs1A |  |  |
| 1no5B |  |  |  |  |  | 2gf4A | 2ewgB |  |  |
| 2imzA |  |  |  |  |  | 3g0kA | 3cvjA |  |  |
| 1yn4A |  |  |  |  |  | 3fhaA | 2hpkA |  |  |
| 4bmbA |  |  |  |  |  | 2aprA | 4fvaC |  |  |
| 1xovA |  |  |  |  |  | 3qmnE | 4lplA |  |  |
| 3ux3B |  |  |  |  |  | 2wynA | 4jx0B |  |  |
| 1gr0A |  |  |  |  |  | 2bo2A | 1zn2A |  |  |
| 2p9xD |  |  |  |  |  | 3km5A | 4k9qA |  |  |
| 4u3eA |  |  |  |  |  | 1qhdA | 1yu4B |  |  |
| 4fgmA |  |  |  |  |  | 1tw8A | 1a77A |  |  |
| 1y7wA |  |  |  |  |  | 2qeaA | 4b3tC |  |  |
| 4iqrF |  |  |  |  |  | 3dd4A | 2jihB |  |  |
| 2x4kB |  |  |  |  |  | 4eoyA | 2z4tA |  |  |
| 3l7xA |  |  |  |  |  | 2vyoA | 3vzdC |  |  |
| 3f3qA |  |  |  |  |  | 2aorB | 2hfkA |  |  |
| 3uk3D |  |  |  |  |  | 3bvcA | 2prnA |  |  |
| 1cw0A |  |  |  |  |  | 4plmA | 1izcA |  |  |
| 3u5gf |  |  |  |  |  | 2fi1A | 4izkA |  |  |
| 2w3zA |  |  |  |  |  | 3h36A | 4i45A |  |  |
| 2qw5A |  |  |  |  |  | 1v0aA | 1oh9A |  |  |
| 4axlA |  |  |  |  |  | 3gg1B | 3b7wA |  |  |
| 2zumA |  |  |  |  |  | 2ricC | 2gwcF |  |  |
| 3wajA |  |  |  |  |  | 3sonA | 2wkpA |  |  |
| 3nw0A |  |  |  |  |  | 2gseA | 3rf6B |  |  |
| 3k9tA |  |  |  |  |  | 1wy9A | 2yfdA |  |  |
| 4dduA |  |  |  |  |  | 1pocA | 3rgnA |  |  |
| 1zmeD |  |  |  |  |  | 1glgA | 3viuA |  |  |
| 4c98A |  |  |  |  |  | 4ozgC | 3pffA |  |  |
| 4gslA |  |  |  |  |  | 1y10C | 4pu5A |  |  |
| 1ue1B |  |  |  |  |  | 4npkA | 3mqyA |  |  |
| 1m55B |  |  |  |  |  | 2v73A | 3i9gL |  |  |
| 2x3bA |  |  |  |  |  | 3pk0C | 2reuA |  |  |
| 3cjpA |  |  |  |  |  | 3trpA | 4e89A |  |  |
| 4hcgA |  |  |  |  |  | 3pf2A | 3gqcB |  |  |
| 4iaoB |  |  |  |  |  | 2vptA | 3e5hA |  |  |
| 2ra6D |  |  |  |  |  | 1dfmA | 2eb6A |  |  |
| 2yikA |  |  |  |  |  | 1nw1A | 2iciA |  |  |
| 4lmyB |  |  |  |  |  | 1ag9B | 2q66A |  |  |
| 1bp3B |  |  |  |  |  | 4gsqA | 4cidA |  |  |
| 7mdhB |  |  |  |  |  | 1rdrA | 2ejqB |  |  |
| 3mz8B |  |  |  |  |  | 4kkiA | 4g75A |  |  |
| 2xpyA |  |  |  |  |  | 4o5mA | 1xtjA |  |  |
| 2w22A |  |  |  |  |  | 2z6iB | 4kjgA |  |  |
| 1p91A |  |  |  |  |  | 3ectA | 4cltA |  |  |
| 1x1cA |  |  |  |  |  | 4aq1A | 4l0jA |  |  |
| 4l8hB |  |  |  |  |  | 2d3dA | 4ml9A |  |  |
| 3fvyA |  |  |  |  |  | 4mlzB | 3ucwD |  |  |
| 3m3bA |  |  |  |  |  | 3bx1C | 2j4eD |  |  |
| 4bhwB |  |  |  |  |  | 3p6bB | 3rcyF |  |  |
| 1twfA |  |  |  |  |  | 3v64A | 3lgyA |  |  |
| 1uvqB |  |  |  |  |  | 3haiA | 4ja9A |  |  |
| 2di3A |  |  |  |  |  | 4g26A | 4cyjB |  |  |
| 1i3oE |  |  |  |  |  | 2o18C | 3pnlA |  |  |
| 4b56A |  |  |  |  |  | 3achA | 2yqcA |  |  |
| 4esnA |  |  |  |  |  | 4cu9B | 3d19A |  |  |
| 1u9kB |  |  |  |  |  | 2qngA | 1fi1A |  |  |
| 4ci1B |  |  |  |  |  | 2zahC | 3shsA |  |  |
| 3w20A |  |  |  |  |  | 3m1rD | 2nogA |  |  |
| 3m4rA |  |  |  |  |  | 3p2yA | 3rnlA |  |  |
| 2a2iA |  |  |  |  |  | 1ydyB | 3cmeC |  |  |
| 4ljoA |  |  |  |  |  | 4mmhA | 2r7dA |  |  |
| 2zktB |  |  |  |  |  | 4l2eA | 2b0tA |  |  |
| 1v15B |  |  |  |  |  | 2nvoA | 3fr8B |  |  |
| 3ubfA |  |  |  |  |  | 1wadA | 2e89C |  |  |
| 1v33A |  |  |  |  |  | 3lvtA | 4b3mM |  |  |
| 3d2nA |  |  |  |  |  | 3aagA | 2ibpB |  |  |
| 3u5ij |  |  |  |  |  | 2qltA | 4at8A |  |  |
| 4h9dA |  |  |  |  |  | 4apxB | 1k4iA |  |  |
| 2yx1A |  |  |  |  |  | 3mt5A | 4ocpA |  |  |
| 2innA |  |  |  |  |  | 1cvrA | 4ba9F |  |  |
| 1uqwB |  |  |  |  |  | 2jcgA | 2q5lA |  |  |
| 3gshA |  |  |  |  |  | 3sh5A | 4lgyA |  |  |
| 2gwgA |  |  |  |  |  | 3u43B | 3btxA |  |  |
| 2a0bA |  |  |  |  |  | 3kg6A | 3q4oA |  |  |
| 4txdA |  |  |  |  |  | 4mlcA | 3raeB |  |  |
| 3dxjD |  |  |  |  |  | 1nrwA | 4lnjA |  |  |
| 3kdsE |  |  |  |  |  | 3db7A | 4kysA |  |  |
| 4cosA |  |  |  |  |  | 4jglA | 3adcA |  |  |
| 2yhgA |  |  |  |  |  | 3ju7B | 3vkgA |  |  |
| 1jccC |  |  |  |  |  | 4mzaB | 3ugvF |  |  |
| 2ze7A |  |  |  |  |  | 1eptB | 3nl6C |  |  |
| 2e26A |  |  |  |  |  | 3qqzA | 4knvA |  |  |
| 3k6iA |  |  |  |  |  | 2gxsB | 4p02B |  |  |
| 4emeC |  |  |  |  |  | 4mixB | 4a35A |  |  |
| 4aycA |  |  |  |  |  | 4ku9A | 4f8bB |  |  |
| 4mo1A |  |  |  |  |  | 1z0bA | 2c71A |  |  |
| 1mzbA |  |  |  |  |  | 3kqgA | 3fpaA |  |  |
| 4p36A |  |  |  |  |  | 3e3rA | 1fxxA |  |  |
| 1dosB |  |  |  |  |  | 4jwpA | 3vr6B |  |  |
| 3s2mA |  |  |  |  |  | 3cjyA | 4mn3A |  |  |
| 4e5vA |  |  |  |  |  | 2vmhA | 1xd3A |  |  |
| 2pebB |  |  |  |  |  | 1srrC | 3t99A |  |  |
| 3lkvA |  |  |  |  |  | 1gn1F | 4p32B |  |  |
| 2hbmA |  |  |  |  |  | 1wkyA | 2c43A |  |  |
| 2ooiA |  |  |  |  |  | 3bowA | 4ogeA |  |  |
| 2agzH |  |  |  |  |  | 3q3qA | 4afyB |  |  |
| 3kaoA |  |  |  |  |  | 2yayA | 1qr0A |  |  |
| 3pw3A |  |  |  |  |  | 2rldC | 2wagA |  |  |
| 2x98A |  |  |  |  |  | 2i1qA | 1t0fA |  |  |
| 3pfeA |  |  |  |  |  | 4iu2B | 3jvvC |  |  |
| 2f6sB |  |  |  |  |  | 2nxpA | 2o1xB |  |  |
| 4mb7A |  |  |  |  |  | 4mkfB | 3f1fK |  |  |
| 3o94B |  |  |  |  |  | 1dl2A | 4iefH |  |  |
| 3ebeC |  |  |  |  |  | 1mnzA | 4anjA |  |  |
| 3obcA |  |  |  |  |  | 2wfkA | 1f6tC |  |  |
| 2ab4A |  |  |  |  |  | 3qtaA | 4kh4B |  |  |
| 1ocyA |  |  |  |  |  | 3asiA | 4fi1A |  |  |
| 3rpdA |  |  |  |  |  | 2xwgB | 4r02V |  |  |
| 1pzwA |  |  |  |  |  | 2hnfA | 4nnaA |  |  |
| 3dxsX |  |  |  |  |  | 5chyA | 2v9jE |  |  |
| 3g0zA |  |  |  |  |  | 2dsoC | 1ka2A |  |  |
| 2qt3A |  |  |  |  |  | 2hq8B | 4nehA |  |  |
| 1yj0A |  |  |  |  |  | 3zukB | 3to3B |  |  |
| 3ntyA |  |  |  |  |  | 3ukgA | 2fa9B |  |  |
| 2qquA |  |  |  |  |  | 1f4nA | 1do0B |  |  |
| 3epmA |  |  |  |  |  | 2e85B | 4ix5B |  |  |
| 3hxsA |  |  |  |  |  | 2bngB | 2z51A |  |  |
| 4ixjB |  |  |  |  |  | 3hjrA | 3l9bA |  |  |
| 3vhtB |  |  |  |  |  | 3lcpD | 4olsD |  |  |
| 4k2wB |  |  |  |  |  | 4b7uA | 3hwoA |  |  |
| 1yixB |  |  |  |  |  | 4ptfA | 2hpiA |  |  |
| 4mahA |  |  |  |  |  | 1o5kA | 4fm9A |  |  |
| 1nvbB |  |  |  |  |  | 4d90A | 1vc9A |  |  |
| 2qm1B |  |  |  |  |  | 3wmwB | 4jrnA |  |  |
| 3tn1A |  |  |  |  |  | 3kptB | 3epsA |  |  |
| 3ce2A |  |  |  |  |  | 3lnpA | 3chmA |  |  |
| 2pajA |  |  |  |  |  | 3rzaA | 1repC |  |  |
| 3pfoB |  |  |  |  |  | 3iprF | 4h9dB |  |  |
| 4nqyA |  |  |  |  |  | 1r17A | 4g7eB |  |  |
| 2vh9B |  |  |  |  |  | 3s55D | 2f7tA |  |  |
| 3eerA |  |  |  |  |  | 2qv6A | 3qqvA |  |  |
| 2oogD |  |  |  |  |  | 1yu0A | 3ddvB |  |  |
| 2d3kB |  |  |  |  |  | 1y1xA | 1vr0A |  |  |
| 3u24A |  |  |  |  |  | 3hx6A | 3vb9B |  |  |
| 3psqA |  |  |  |  |  | 4iu2A | 3dtyE |  |  |
| 1y02A |  |  |  |  |  | 3zm8A | 4ep4B |  |  |
| 4ao7A |  |  |  |  |  | 4ht4A | 2i6kA |  |  |
| 4dguA |  |  |  |  |  | 3votA | 3cisB |  |  |
| 3na7A |  |  |  |  |  | 3b40A | 1t8qB |  |  |
| 1v47A |  |  |  |  |  | 1f0oA | 2xs4A |  |  |
| 2y0oA |  |  |  |  |  | 2osdA | 4kftA |  |  |
| 2cuaB |  |  |  |  |  | 2jbhB | 1hbnA |  |  |
| 2xnjA |  |  |  |  |  | 1of3A | 4hvyA |  |  |
| 3tj9A |  |  |  |  |  | 2eabA | 3f1yA |  |  |
| 2djwB |  |  |  |  |  | 4i9wA | 1n52A |  |  |
| 2j13A |  |  |  |  |  | 1lqvB | 2jd5A |  |  |
| 2ceyA |  |  |  |  |  | 2snsA | 2c2jA |  |  |
| 4o5oB |  |  |  |  |  | 3hciB | 4jzvA |  |  |
| 3tasB |  |  |  |  |  | 3vm5A | 1iovA |  |  |
| 1rmdA |  |  |  |  |  | 3pgsA | 1inpA |  |  |
| 3o2gA |  |  |  |  |  | 3b3dA | 2hmaA |  |  |
| 3nvoA |  |  |  |  |  | 1p49A | 3hujH |  |  |
| 3t8wA |  |  |  |  |  | 1c1yB | 2yl6A |  |  |
| 4hk5D |  |  |  |  |  | 4eg9A | 3c8cB |  |  |
| 4egeA |  |  |  |  |  | 3f8hA | 2zpuA |  |  |
| 2z45A |  |  |  |  |  | 2fpwB | 4gi2A |  |  |
| 4e45E |  |  |  |  |  | 3djlA | 3qurA |  |  |
| 3lotD |  |  |  |  |  | 4hvkA | 3hy6A |  |  |
| 3eefA |  |  |  |  |  | 2ddfA | 4py5A |  |  |
| 1yw4B |  |  |  |  |  | 2arhA | 1gs6X |  |  |
| 3lnnA |  |  |  |  |  | 3kmvE | 4il8A |  |  |
| 3swnQ |  |  |  |  |  | 3wmtA | 4kp1A |  |  |
| 2qnwA |  |  |  |  |  | 4p5eB | 4ex4B |  |  |
| 3st7A |  |  |  |  |  | 3ltlA | 4orkA |  |  |
| 2qtvA |  |  |  |  |  | 4mn0A | 2qdfA |  |  |
| 2d74B |  |  |  |  |  | 2x7qA | 4bjuA |  |  |
| 4fmuA |  |  |  |  |  | 3ojyB | 2vosA |  |  |
| 3c5kA |  |  |  |  |  | 1ng0C | 3feuA |  |  |
| 1vshA |  |  |  |  |  | 1nenB | 1n2gB |  |  |
| 4eogA |  |  |  |  |  | 2xmrA | 3auyA |  |  |
| 4ebbA |  |  |  |  |  | 1zmrA | 2ox6A |  |  |
| 3ee6A |  |  |  |  |  | 3njhD | 3dv9A |  |  |
| 2vqjA |  |  |  |  |  | 2eslF | 4oxdA |  |  |
| 3ax1A |  |  |  |  |  | 3ffkA | 3cuzA |  |  |
| 3cxlA |  |  |  |  |  | 2wfrA | 2bonA |  |  |
| 2uurA |  |  |  |  |  | 1fwxA | 2hmcA |  |  |
| 3tc8A |  |  |  |  |  | 4gz9A | 2pyjB |  |  |
| 3kflA |  |  |  |  |  | 1k6sB | 3l12A |  |  |
| 1xc3A |  |  |  |  |  | 1g5cA | 4jndA |  |  |
| 4l58A |  |  |  |  |  | 3godA | 1gxbD |  |  |
| 4bt2A |  |  |  |  |  | 1sumB | 4bebB |  |  |
| 2zzwA |  |  |  |  |  | 3v20A | 1v98A |  |  |
| 4ay8A |  |  |  |  |  | 3lwaA | 3rvpB |  |  |
| 2uz9A |  |  |  |  |  | 3u16B | 3fxgE |  |  |
| 2au3A |  |  |  |  |  | 1fsuA | 1qhaA |  |  |
| 3st1A |  |  |  |  |  | 1ej8A | 3bc1A |  |  |
| 1jocB |  |  |  |  |  | 4dokA | 2b06A |  |  |
| 3umiA |  |  |  |  |  | 3k28A | 1m0wA |  |  |
| 2ac3A |  |  |  |  |  | 1h30A | 3t2dA |  |  |
| 3favB |  |  |  |  |  | 3fspA | 1waxA |  |  |
| 2zetC |  |  |  |  |  | 2iunB | 3htwA |  |  |
| 1gudA |  |  |  |  |  | 1e7mA | 2xzwC |  |  |
| 1q14A |  |  |  |  |  | 3whnB | 2ouxA |  |  |
| 4hl2A |  |  |  |  |  | 1kp4A | 4odjA |  |  |
| 4e4wB |  |  |  |  |  | 4il1A | 4kvaB |  |  |
| 3alrC |  |  |  |  |  | 3by9A | 2ou7A |  |  |
| 3i3wA |  |  |  |  |  | 1h6xA | 4m0lC |  |  |
| 3mvqF |  |  |  |  |  | 3k5mA | 3eafA |  |  |
| 4areA |  |  |  |  |  | 2iiiA | 3chlA |  |  |
| 2oo4A |  |  |  |  |  | 1w3bA | 4iynA |  |  |
| 2rknA |  |  |  |  |  | 3im9A | 3tzfA |  |  |
| 2xqcD |  |  |  |  |  | 4hojA | 4kjcT |  |  |
| 2nsfA |  |  |  |  |  | 3oyrB | 4gwiA |  |  |
| 4ewqA |  |  |  |  |  | 1mu5A | 3sy8B |  |  |
| 4q7rA |  |  |  |  |  | 1tn3A | 1u5rB |  |  |
| 3kj1A |  |  |  |  |  | 2y8kA | 2uagA |  |  |
| 2gb5A |  |  |  |  |  | 1j1tA | 4hizB |  |  |
| 3vusA |  |  |  |  |  | 3mseB | 1m74A |  |  |
| 2j4xA |  |  |  |  |  | 2g8sA | 1vbgA |  |  |
| 2hc9A |  |  |  |  |  | 1bf2A | 4eenA |  |  |
| 1nltA |  |  |  |  |  | 2x8hA | 3kb4B |  |  |
| 3b0aE |  |  |  |  |  | 3dtnA | 2is4A |  |  |
| 3kl7A |  |  |  |  |  | 1oflA | 4czkA |  |  |
| 2aydA |  |  |  |  |  | 1m5oF | 3ea0B |  |  |
| 3l1eA |  |  |  |  |  | 2tbvC | 1gsaA |  |  |
| 3u5ga |  |  |  |  |  | 2pagA | 2wcjA |  |  |
| 2qswA |  |  |  |  |  | 3dzcA | 2x5fB |  |  |
| 4b6zB |  |  |  |  |  | 2i7aA | 1wc1A |  |  |
| 4ngeD |  |  |  |  |  | 3s6pC | 3rgwL |  |  |
| 1xocA |  |  |  |  |  | 3eqiA | 3qe5A |  |  |
| 3i2dA |  |  |  |  |  | 4uojB | 3shqA |  |  |
| 3mhhE |  |  |  |  |  | 3pe7A | 1wywA |  |  |
| 4kujB |  |  |  |  |  | 1nl1A | 2ddxA |  |  |
| 1q9uB |  |  |  |  |  | 1tvgA | 3k1uA |  |  |
| 4cn9A |  |  |  |  |  | 1n7sB | 1yrbB |  |  |
| 4ewlA |  |  |  |  |  | 4ll5A | 1rypH |  |  |
| 2oikC |  |  |  |  |  | 4lp7D | 3g3nA |  |  |
| 4ne7A |  |  |  |  |  | 1j24A | 4oojC |  |  |
| 3dciA |  |  |  |  |  | 4aejB | 3fdoA |  |  |
| 1nuiA |  |  |  |  |  | 3zzrA | 3tebA |  |  |
| 2y2bB |  |  |  |  |  | 4ollA | 1qlgA |  |  |
| 2hbaA |  |  |  |  |  | 1zdeA | 2w5aA |  |  |
| 3bliA |  |  |  |  |  | 4dsiA | 3ir2A |  |  |
| 3mcxA |  |  |  |  |  | 1f8vA | 4dbhA |  |  |
| 3qz6A |  |  |  |  |  | 4m5vA | 2onsA |  |  |
| 4fxoB |  |  |  |  |  | 3mdoA | 1nhiA |  |  |
| 3iehA |  |  |  |  |  | 4be6B | 1yvpB |  |  |
| 4ggjA |  |  |  |  |  | 3ntlB | 2a5yC |  |  |
| 3rcmA |  |  |  |  |  | 1zobA | 4twkA |  |  |
| 2w9nA |  |  |  |  |  | 3qdnA | 3b9tC |  |  |
| 2cjsC |  |  |  |  |  | 2b50B | 3m7kA |  |  |
| 3f2bA |  |  |  |  |  | 3svlB | 3hs0I |  |  |
| 4qhiB |  |  |  |  |  | 2wiiA | 3h70A |  |  |
| 2o6pA |  |  |  |  |  | 3p4gB | 4f0yB |  |  |
| 1jazA |  |  |  |  |  | 3srgA | 3mbiA |  |  |
| 3nitA |  |  |  |  |  | 4iaiA | 2wl9A |  |  |
| 1oahA |  |  |  |  |  | 3us9A | 3opkC |  |  |
| 4p53A |  |  |  |  |  | 4nwoA | 4cejB |  |  |
| 1u5kA |  |  |  |  |  | 2v3tB | 3vthB |  |  |
| 2y7iA |  |  |  |  |  | 3ibzA | 2xd4A |  |  |
| 2ph1A |  |  |  |  |  | 3r5xD | 3ovbA |  |  |
| 3kfuF |  |  |  |  |  | 3kezB | 4ku9A |  |  |
| 2ou3B |  |  |  |  |  | 4n5xA | 4cnsD |  |  |
| 1vs0B |  |  |  |  |  | 1u94A | 4l39A |  |  |
| 4d09A |  |  |  |  |  | 3e03A | 3c15A |  |  |
| 4axvA |  |  |  |  |  | 3n1uA | 1cx0A |  |  |
| 1urjB |  |  |  |  |  | 2w27A | 2bgwA |  |  |
| 2cbiB |  |  |  |  |  | 2ydxA | 1hk7A |  |  |
| 3ephA |  |  |  |  |  | 2xecB | 3iilA |  |  |
| 3d1tA |  |  |  |  |  | 4iauA | 3fr6A |  |  |
| 4hccA |  |  |  |  |  | 1y7uA | 3akeA |  |  |
| 1v72A |  |  |  |  |  | 4fzmA | 3no1C |  |  |
| 4mz7B |  |  |  |  |  | 3pgvA | 3p96A |  |  |
| 2vacA |  |  |  |  |  | 2yf1A | 2vd9A |  |  |
| 1eteA |  |  |  |  |  | 1vsiA | 4fmmB |  |  |
| 3vpbB |  |  |  |  |  | 4c0kA | 1fvuD |  |  |
| 3t9oB |  |  |  |  |  | 3n9kA | 2qs8A |  |  |
| 1xkiA |  |  |  |  |  | 4b60B | 3n6qD |  |  |
| 4kavA |  |  |  |  |  | 4kpoA | 1tw1A |  |  |
| 2f44A |  |  |  |  |  | 1tadC | 4oauC |  |  |
| 3iuuA |  |  |  |  |  | 1rq5A | 2fyrA |  |  |
| 4au7A |  |  |  |  |  | 3gw6F | 2a0iA |  |  |
| 3iciA |  |  |  |  |  | 1je5B | 2eb1A |  |  |
| 4jd1B |  |  |  |  |  | 1heiA | 3st8A |  |  |
| 1uwyA |  |  |  |  |  | 2f1wA | 4dmbB |  |  |
| 3sngA |  |  |  |  |  | 3kaaB | 2z4sA |  |  |
| 3lecA |  |  |  |  |  | 3tbdA | 2cjaB |  |  |
| 2aqsA |  |  |  |  |  | 2gskA | 3wcaB |  |  |
| 3av4A |  |  |  |  |  | 4awdA | 1khzB |  |  |
| 1zbdB |  |  |  |  |  | 2q04A | 3b1rD |  |  |
| 3htkC |  |  |  |  |  | 2uvpC | 3wagA |  |  |
| 1eucB |  |  |  |  |  | 4lt6A | 2eh3A |  |  |
| 2w0mA |  |  |  |  |  | 2y3nB | 2qt0A |  |  |
| 3mbjA |  |  |  |  |  | 1uocA | 2guhA |  |  |
| 2rc6A |  |  |  |  |  | 2h9dD | 1y23B |  |  |
| 2hjnA |  |  |  |  |  | 2cftA | 2h28A |  |  |
| 4binA |  |  |  |  |  | 2h0bC | 3iukA |  |  |
| 1qwrA |  |  |  |  |  | 1su3B | 4fypA |  |  |
| 2dxnB |  |  |  |  |  | 3rrvB | 3umbA |  |  |
| 2wvkA |  |  |  |  |  | 2omxA | 3bp1D |  |  |
| 2owaA |  |  |  |  |  | 2g8kA | 3nx3A |  |  |
| 3t92A |  |  |  |  |  | 4ovyA | 3c9hB |  |  |
| 2vz5A |  |  |  |  |  | 2ydpA | 3kd5E |  |  |
| 1q2lA |  |  |  |  |  | 3ppvA | 1sh3B |  |  |
| 3m1nB |  |  |  |  |  | 2xonL | 4d26A |  |  |
| 3aalA |  |  |  |  |  | 4h2aA | 3d2fA |  |  |
| 4hvlA |  |  |  |  |  | 2ww8A | 4n0nA |  |  |
| 1lbaA |  |  |  |  |  | 3eu3A | 4n5vB |  |  |
| 4lj0A |  |  |  |  |  | 3s82B | 1zshA |  |  |
| 3vpeA |  |  |  |  |  | 1hcuB | 4q7fA |  |  |
| 2gu1A |  |  |  |  |  | 2xrcB | 3wkyB |  |  |
| 2eh9A |  |  |  |  |  | 4g97A | 4l87A |  |  |
| 1bp3A |  |  |  |  |  | 2a11A | 2xamB |  |  |
| 2wc8D |  |  |  |  |  | 4fhpA | 3k4zA |  |  |
| 1d9xA |  |  |  |  |  | 3qsjA | 4m9qB |  |  |
| 2fygA |  |  |  |  |  | 1xkdA | 4eruA |  |  |
| 4i11A |  |  |  |  |  | 1e3aB | 3kylA |  |  |
| 2pw6A |  |  |  |  |  | 4nhfF | 3v9xB |  |  |
| 4hd5A |  |  |  |  |  | 3vppA | 3kb2B |  |  |
| 3ibmB |  |  |  |  |  | 3gbhB | 3tdwA |  |  |
| 4ogeA |  |  |  |  |  | 3riwB | 4hubY |  |  |
| 1czfB |  |  |  |  |  | 2cy5A | 1id0A |  |  |
| 4k25A |  |  |  |  |  | 1pmjX | 3t1oA |  |  |
| 3lmcA |  |  |  |  |  | 2ezvB | 2ddoA |  |  |
| 2j7jA |  |  |  |  |  | 3mvsA | 4kg0A |  |  |
| 3s6lD |  |  |  |  |  | 2bf0X | 3dxjB |  |  |
| 4gxwA |  |  |  |  |  | 2bkoA | 4qhzD |  |  |
| 3hkoA |  |  |  |  |  | 4i9xB | 2zdrA |  |  |
| 2x4hC |  |  |  |  |  | 2ygpA | 2j4hB |  |  |
| 1pb0A |  |  |  |  |  | 3nsjA | 1c9kC |  |  |
| 2wazX |  |  |  |  |  | 1jhnA | 2x5zA |  |  |
| 3bkfA |  |  |  |  |  | 2imwP | 1e0jB |  |  |
| 1x3wA |  |  |  |  |  | 4i4tA | 3e7eA |  |  |
| 3fggA |  |  |  |  |  | 1ex0B | 4m8oA |  |  |
| 1vddA |  |  |  |  |  | 4iejA | 3d2jA |  |  |
| 2pkpA |  |  |  |  |  | 4m00A | 3hhiA |  |  |
| 1vecA |  |  |  |  |  | 2bw1K | 3fesA |  |  |
| 2ogjA |  |  |  |  |  | 2nq6A | 2zxrA |  |  |
| 1eu4A |  |  |  |  |  | 3o78A | 3melC |  |  |
| 2w4lA |  |  |  |  |  | 4kywA | 3h4sA |  |  |
| 4i6vB |  |  |  |  |  | 3afgB | 3au0A |  |  |
| 1pguB |  |  |  |  |  | 3d1rA | 4h16A |  |  |
| 2xb4A |  |  |  |  |  | 2g18D | 3ibsA |  |  |
| 1vk9A |  |  |  |  |  |  | 4af5A |  |  |
| 3sp7A |  |  |  |  |  |  | 1txuA |  |  |
| 3o9pA |  |  |  |  |  |  | 3keoB |  |  |
| 3iu6A |  |  |  |  |  |  | 3nz2C |  |  |
| 2x5rA |  |  |  |  |  |  | 1fgsA |  |  |
| 3b2zF |  |  |  |  |  |  | 3d6aA |  |  |
| 4bs9A |  |  |  |  |  |  | 4iikA |  |  |
| 3l0aA |  |  |  |  |  |  | 4f9aC |  |  |
| 1umyD |  |  |  |  |  |  | 4gyiA |  |  |
| 2yr2B |  |  |  |  |  |  | 1tkdA |  |  |
| 4gwmA |  |  |  |  |  |  | 1lp1B |  |  |
| 4c2mB |  |  |  |  |  |  | 1mxgA |  |  |
| 4g1pA |  |  |  |  |  |  | 3uwkA |  |  |
| 2rhkC |  |  |  |  |  |  | 3vmmA |  |  |
| 1mwqA |  |  |  |  |  |  | 3lghA |  |  |
| 4n0lA |  |  |  |  |  |  | 3g2fA |  |  |
| 4lzuA |  |  |  |  |  |  | 3f1fT |  |  |
| 3gglA |  |  |  |  |  |  | 3g1cA |  |  |
| 1jqgA |  |  |  |  |  |  | 4ifdJ |  |  |
| 3u9gA |  |  |  |  |  |  | 1lm3B |  |  |
| 1odzB |  |  |  |  |  |  | 4cgkB |  |  |
| 4n4wA |  |  |  |  |  |  | 3zc0B |  |  |
| 4m6gA |  |  |  |  |  |  | 4q57B |  |  |
| 1dy1A |  |  |  |  |  |  | 2yjgA |  |  |
| 3hphC |  |  |  |  |  |  | 3f1tB |  |  |
| 3lvzB |  |  |  |  |  |  | 2qg8A |  |  |
| 2hjgA |  |  |  |  |  |  | 4g9bA |  |  |
| 4dyuH |  |  |  |  |  |  | 3e54B |  |  |
| 4ii1B |  |  |  |  |  |  | 1k6dB |  |  |
| 2xz0B |  |  |  |  |  |  | 2zxuB |  |  |
| 1yewE |  |  |  |  |  |  | 3mwcA |  |  |
| 2bjrA |  |  |  |  |  |  | 3ckgA |  |  |
| 1cjvA |  |  |  |  |  |  | 3n7xA |  |  |
| 4bf7A |  |  |  |  |  |  | 3nc3B |  |  |
| 3m4wA |  |  |  |  |  |  | 3flkC |  |  |
| 3l22A |  |  |  |  |  |  | 3cg4A |  |  |
| 1kl9A |  |  |  |  |  |  | 4h2uB |  |  |
| 2g54A |  |  |  |  |  |  | 2izoC |  |  |
| 2e46A |  |  |  |  |  |  | 4cw7H |  |  |
| 2apoB |  |  |  |  |  |  | 3w15A |  |  |
| 1cvrA |  |  |  |  |  |  | 1mogA |  |  |
| 1pwuA |  |  |  |  |  |  | 1tk6C |  |  |
| 3swfB |  |  |  |  |  |  | 2wefA |  |  |
| 2z00A |  |  |  |  |  |  | 3s70A |  |  |
| 3l9wB |  |  |  |  |  |  | 2dlcX |  |  |
| 1x0tA |  |  |  |  |  |  | 1agrA |  |  |
| 3vk6A |  |  |  |  |  |  | 3zdbA |  |  |
| 1btkB |  |  |  |  |  |  | 1hyoB |  |  |
| 4i2fA |  |  |  |  |  |  | 4bhqA |  |  |
| 1wkqA |  |  |  |  |  |  | 1slhC |  |  |
| 1py0A |  |  |  |  |  |  | 1mauA |  |  |
| 1k24A |  |  |  |  |  |  | 9rubA |  |  |
| 1p9wA |  |  |  |  |  |  | 3e2vA |  |  |
| 3chvA |  |  |  |  |  |  | 3ec2A |  |  |
| 1ryqA |  |  |  |  |  |  | 4gp2A |  |  |
| 1vliA |  |  |  |  |  |  | 3twbC |  |  |
| 1b0nA |  |  |  |  |  |  | 1xedA |  |  |
| 3ta7A |  |  |  |  |  |  | 3akkA |  |  |
| 4n0nA |  |  |  |  |  |  | 2b56A |  |  |
| 3cosD |  |  |  |  |  |  | 3kgxA |  |  |
| 4aojC |  |  |  |  |  |  | 2dqbC |  |  |
| 4ac1X |  |  |  |  |  |  | 3s5mA |  |  |
| 3rzaA |  |  |  |  |  |  | 2iiiA |  |  |
| 3dcpA |  |  |  |  |  |  | 1ii0B |  |  |
| 4ct0A |  |  |  |  |  |  | 2j7qA |  |  |
| 1s1gB |  |  |  |  |  |  | 4gdzA |  |  |
| 1r5tD |  |  |  |  |  |  | 1ozgA |  |  |
| 4mhnA |  |  |  |  |  |  | 2jk1A |  |  |
| 4iuwA |  |  |  |  |  |  | 1xu4A |  |  |
| 2ixdA |  |  |  |  |  |  | 1xz8A |  |  |
| 1ck1A |  |  |  |  |  |  | 3sb5A |  |  |
| 3mwmA |  |  |  |  |  |  | 2h5nA |  |  |
| 3r2jB |  |  |  |  |  |  | 2bifA |  |  |
| 4m8oA |  |  |  |  |  |  | 4b43A |  |  |
| 2qhaA |  |  |  |  |  |  | 3sh1C |  |  |
| 1l7oB |  |  |  |  |  |  | 2g9zA |  |  |
| 3n71A |  |  |  |  |  |  | 4biwA |  |  |
| 2i13A |  |  |  |  |  |  | 3lijA |  |  |
| 1pq4B |  |  |  |  |  |  | 3tptB |  |  |
| 4nm6A |  |  |  |  |  |  | 2pkeA |  |  |
| 2riqA |  |  |  |  |  |  | 4i4tF |  |  |
| 4e6rA |  |  |  |  |  |  | 3crlB |  |  |
| 2dh3A |  |  |  |  |  |  | 1vsdA |  |  |
| 2fpwB |  |  |  |  |  |  | 4ayxA |  |  |
| 1txlA |  |  |  |  |  |  | 4ofzA |  |  |
| 1auiA |  |  |  |  |  |  | 2v0nA |  |  |
| 3fymA |  |  |  |  |  |  | 4o2bA |  |  |
| 1vykA |  |  |  |  |  |  | 3ai9X |  |  |
| 3vrhA |  |  |  |  |  |  | 2ceaC |  |  |
| 2e7yA |  |  |  |  |  |  | 3qbmA |  |  |
| 4ndhB |  |  |  |  |  |  | 1qs0A |  |  |
| 4ifdJ |  |  |  |  |  |  | 4p0pA |  |  |
| 2xigC |  |  |  |  |  |  | 1pt6B |  |  |
| 1z2lA |  |  |  |  |  |  | 2zy9B |  |  |
| 4oteB |  |  |  |  |  |  | 3eqiA |  |  |
| 3giuB |  |  |  |  |  |  | 4i0uF |  |  |
| 4nl4H |  |  |  |  |  |  | 3al5B |  |  |
| 2czrA |  |  |  |  |  |  | 2hx0A |  |  |
| 2izoC |  |  |  |  |  |  | 2j17B |  |  |
| 4b9pA |  |  |  |  |  |  | 2vd3A |  |  |
| 3idvA |  |  |  |  |  |  | 1ezwA |  |  |
| 3go9A |  |  |  |  |  |  | 3c5hA |  |  |
| 1ve0A |  |  |  |  |  |  | 3fm5C |  |  |
| 3ivbA |  |  |  |  |  |  | 3tlxA |  |  |
| 3tp2A |  |  |  |  |  |  | 2xbuB |  |  |
| 4f6nA |  |  |  |  |  |  | 2qb8A |  |  |
| 3rhgA |  |  |  |  |  |  | 1g9gA |  |  |
| 1eb6A |  |  |  |  |  |  | 4j7lA |  |  |
| 4aapA |  |  |  |  |  |  | 2ap9A |  |  |
| 2v8lA |  |  |  |  |  |  | 3vmqA |  |  |
| 3lubF |  |  |  |  |  |  | 1ecbC |  |  |
| 3m1mA |  |  |  |  |  |  | 4dpgB |  |  |
| 2cyeC |  |  |  |  |  |  | 2d0oA |  |  |
| 3h50A |  |  |  |  |  |  | 3crcB |  |  |
| 3s5mA |  |  |  |  |  |  | 3fpiA |  |  |
| 2qkdA |  |  |  |  |  |  | 1d3yB |  |  |
| 2d74A |  |  |  |  |  |  | 3o61D |  |  |
| 4c9zA |  |  |  |  |  |  | 3qnmA |  |  |
| 2c5wB |  |  |  |  |  |  | 4ksiA |  |  |
| 2hu9A |  |  |  |  |  |  | 3jwgA |  |  |
| 1lmlA |  |  |  |  |  |  | 3iivB |  |  |
| 1rfuF |  |  |  |  |  |  | 4a7zA |  |  |
| 2eerA |  |  |  |  |  |  | 2yc4C |  |  |
| 1xagA |  |  |  |  |  |  | 2obbA |  |  |
| 4n4rC |  |  |  |  |  |  | 3f2aA |  |  |
| 1yt3A |  |  |  |  |  |  | 4kgqC |  |  |
| 1bi0A |  |  |  |  |  |  | 3gybA |  |  |
| 2imrA |  |  |  |  |  |  | 4ay2A |  |  |
| 2iimA |  |  |  |  |  |  | 3cbgA |  |  |
| 3q4rA |  |  |  |  |  |  | 1rddA |  |  |
| 4iumA |  |  |  |  |  |  | 4f1mA |  |  |
| 4m0cA |  |  |  |  |  |  | 3sahB |  |  |
| 3oa4A |  |  |  |  |  |  | 2vedB |  |  |
| 1r8qE |  |  |  |  |  |  | 3dc7B |  |  |
| 3menB |  |  |  |  |  |  | 4cw7A |  |  |
| 1i7wC |  |  |  |  |  |  | 1ydfA |  |  |
| 3sp4B |  |  |  |  |  |  | 1yd1A |  |  |
| 3ihpB |  |  |  |  |  |  | 2xo6A |  |  |
| 4mt2A |  |  |  |  |  |  | 3b2sA |  |  |
| 2qa1A |  |  |  |  |  |  | 4qcr6 |  |  |
| 3e38B |  |  |  |  |  |  | 4efcA |  |  |
| 4gbmA |  |  |  |  |  |  | 1vs0A |  |  |
| 1z05A |  |  |  |  |  |  | 3ngoA |  |  |
| 3bb6C |  |  |  |  |  |  | 1dj9A |  |  |
| 2xb3A |  |  |  |  |  |  | 4ghlA |  |  |
| 1u8bA |  |  |  |  |  |  | 2gj8D |  |  |
| 1keaA |  |  |  |  |  |  | 2ywrA |  |  |
| 3vuvA |  |  |  |  |  |  | 4gmjB |  |  |
| 3n12A |  |  |  |  |  |  | 1f8iB |  |  |
| 2y6eB |  |  |  |  |  |  | 1j1cB |  |  |
| 3tp9A |  |  |  |  |  |  | 3k4iB |  |  |
| 3gl6A |  |  |  |  |  |  | 3mgaB |  |  |
| 1qrlA |  |  |  |  |  |  | 4ffdA |  |  |
| 1vheA |  |  |  |  |  |  | 4qgpA |  |  |
| 4ox3A |  |  |  |  |  |  | 3vkbB |  |  |
| 2p1hA |  |  |  |  |  |  | 1ksfX |  |  |
| 1t4wA |  |  |  |  |  |  | 3sm4B |  |  |
| 3zg6A |  |  |  |  |  |  | 3i2bA |  |  |
| 3mlpA |  |  |  |  |  |  | 3cmqA |  |  |
| 3ug7A |  |  |  |  |  |  | 3dkxC |  |  |
| 2glzA |  |  |  |  |  |  | 3fhdA |  |  |
| 1su3B |  |  |  |  |  |  | 4cs3A |  |  |
| 2dt8A |  |  |  |  |  |  | 3oyzA |  |  |
| 4ivvA |  |  |  |  |  |  | 1rk2B |  |  |
| 4o6sA |  |  |  |  |  |  | 4lrsA |  |  |
| 1s4iB |  |  |  |  |  |  | 2i89B |  |  |
| 4ccgX |  |  |  |  |  |  | 3p2lA |  |  |
| 2xoeA |  |  |  |  |  |  | 3r7wB |  |  |
| 2xl9B |  |  |  |  |  |  | 1h65A |  |  |
| 3u5ep |  |  |  |  |  |  | 3ug7A |  |  |
| 3h7tA |  |  |  |  |  |  | 4ig8A |  |  |
| 1nyrA |  |  |  |  |  |  | 4k6eA |  |  |
| 2qgsA |  |  |  |  |  |  | 2jcbA |  |  |
| 3tmiA |  |  |  |  |  |  | 4otpA |  |  |
| 1y9qA |  |  |  |  |  |  | 3riiA |  |  |
| 2cjbB |  |  |  |  |  |  | 4dwoA |  |  |
| 4ladB |  |  |  |  |  |  | 3fcsB |  |  |
| 3gs2A |  |  |  |  |  |  | 4j00B |  |  |
| 3o70A |  |  |  |  |  |  | 2aleA |  |  |
| 2wbtA |  |  |  |  |  |  | 2a5lB |  |  |
| 2nvuB |  |  |  |  |  |  | 1nugB |  |  |
| 3ii1A |  |  |  |  |  |  | 2ww8A |  |  |
| 4gy5A |  |  |  |  |  |  | 4ty0A |  |  |
| 3hruA |  |  |  |  |  |  | 2q9pA |  |  |
| 2cd9A |  |  |  |  |  |  | 4k0bB |  |  |
| 2pg3A |  |  |  |  |  |  | 3n4pB |  |  |
| 4ap4A |  |  |  |  |  |  | 2vpnB |  |  |
| 2yjpA |  |  |  |  |  |  | 4bgaC |  |  |
| 3wi9A |  |  |  |  |  |  | 4gt8A |  |  |
| 4kywA |  |  |  |  |  |  | 4hutA |  |  |
| 4cdgA |  |  |  |  |  |  | 1ehiA |  |  |
|  |  |  |  |  |  |  | 4brnA |  |  |
|  |  |  |  |  |  |  | 3hzhA |  |  |
|  |  |  |  |  |  |  | 3gygA |  |  |
|  |  |  |  |  |  |  | 2j0wA |  |  |
|  |  |  |  |  |  |  | 3qtpB |  |  |
|  |  |  |  |  |  |  | 1vmaA |  |  |
|  |  |  |  |  |  |  | 2c9eA |  |  |
|  |  |  |  |  |  |  | 3stpA |  |  |
|  |  |  |  |  |  |  | 4fl2A |  |  |
|  |  |  |  |  |  |  | 3ll6B |  |  |
|  |  |  |  |  |  |  | 1aihC |  |  |
|  |  |  |  |  |  |  | 3k1eB |  |  |
|  |  |  |  |  |  |  | 2bhzA |  |  |
|  |  |  |  |  |  |  | 3sn0A |  |  |
|  |  |  |  |  |  |  | 1ynsA |  |  |
|  |  |  |  |  |  |  | 2fn0B |  |  |
|  |  |  |  |  |  |  | 4a0gB |  |  |
|  |  |  |  |  |  |  | 1f51D |  |  |
|  |  |  |  |  |  |  | 3b5iA |  |  |
|  |  |  |  |  |  |  | 4e4sF |  |  |
|  |  |  |  |  |  |  | 3r09A |  |  |
|  |  |  |  |  |  |  | 3edvB |  |  |
|  |  |  |  |  |  |  | 2ae8B |  |  |
|  |  |  |  |  |  |  | 4k7yA |  |  |
|  |  |  |  |  |  |  | 4ottB |  |  |
|  |  |  |  |  |  |  | 4mndA |  |  |
|  |  |  |  |  |  |  | 1v0dA |  |  |
|  |  |  |  |  |  |  | 2o06A |  |  |
|  |  |  |  |  |  |  | 2vk6A |  |  |
|  |  |  |  |  |  |  | 3cnxC |  |  |
|  |  |  |  |  |  |  | 3lacB |  |  |
|  |  |  |  |  |  |  | 3n1eB |  |  |
|  |  |  |  |  |  |  | 2hcfA |  |  |
